# Dissecting caspase-2-mediated cell death: from intrinsic PIDDosome activation to chemical modulation

**DOI:** 10.1093/procel/pwae020

**Published:** 2024-04-27

**Authors:** Mengxue Zeng, Kun Wang, Qingcui Wu, Jingjin Ding, Dan Xie, Xiangbing Qi, Feng Shao

**Affiliations:** State Key Laboratory of Oncology in South China, Guangdong Provincial Clinical Research Center for Cancer, Sun Yat-sen University Cancer Center, Guangzhou 510060, China; National Institute of Biological Sciences, Beijing, Beijing 102206, China; National Institute of Biological Sciences, Beijing, Beijing 102206, China; National Institute of Biological Sciences, Beijing, Beijing 102206, China; National Institute of Biological Sciences, Beijing, Beijing 102206, China; Key Laboratory of Biomacromolecules (CAS), National Laboratory of Biomacromolecules, CAS Center for Excellence in Biomacromolecules, Institute of Biophysics, Chinese Academy of Sciences, Beijing 100101, China; University of Chinese Academy of Sciences, Beijing 100049, China; State Key Laboratory of Oncology in South China, Guangdong Provincial Clinical Research Center for Cancer, Sun Yat-sen University Cancer Center, Guangzhou 510060, China; National Institute of Biological Sciences, Beijing, Beijing 102206, China; State Key Laboratory of Oncology in South China, Guangdong Provincial Clinical Research Center for Cancer, Sun Yat-sen University Cancer Center, Guangzhou 510060, China; National Institute of Biological Sciences, Beijing, Beijing 102206, China; Key Laboratory of Biomacromolecules (CAS), National Laboratory of Biomacromolecules, CAS Center for Excellence in Biomacromolecules, Institute of Biophysics, Chinese Academy of Sciences, Beijing 100101, China; Research Unit of Pyroptosis and Immunity, Chinese Academy of Medical Sciences and National Institute of Biological Sciences, Beijing, Beijing 102206, China; Changping Laboratory, Beijing 102206, China; Tsinghua Institute of Multidisciplinary Biomedical Research, Tsinghua University, Beijing 102206, China; New Cornerstone Science Laboratory, Shenzhen 518000, China

**Keywords:** caspase-2, PIDDosome, BID, apoptosis, chemical screen, agonist

## Abstract

Caspase-2, a highly conserved member of the caspase family, is considered an initiator caspase that triggers apoptosis in response to some cellular stresses. Previous studies suggest that an intracellular multi-protein complex PIDDosome, induced by genotoxic stress, serves as a platform for caspase-2 activation. Due to caspase-2’s inability to process effector caspases, however, the mechanism underlying caspase-2-mediated cell death upon PIDDosome activation remains unclear. Here, we conducted an unbiased genome-wide genetic screen and identified that the Bcl2 family protein BID is required for PIDDosome-induced, caspase-2-mediated apoptosis. PIDDosome-activated caspase-2 directly and functionally processes BID to signal the mitochondrial pathway for apoptosis induction. In addition, a designed chemical screen identified a compound, HUHS015, which specifically activates caspase-2-mediated apoptosis. HUHS015-stimulated apoptosis also requires BID but is independent of the PIDDosome. Through extensive structure–activity relationship efforts, we identified a derivative with a potency of ~60 nmol/L in activating caspase-2-mediated apoptosis. The HUHS015-series of compounds act as efficient agonists that directly target the interdomain linker in caspase-2, representing a new mode of initiator caspase activation. Human and mouse caspase-2 differ in two crucial residues in the linker, rendering a selectivity of the agonists for human caspase-2. The caspase-2 agonists are valuable tools to explore the physiological roles of caspase-2-mediated cell death and a base for developing small-molecule drugs for relevant diseases.

## Introduction

Caspases are a family of evolutionarily conserved cysteine proteases that play a crucial role in programmed cell death and inflammation. Mammalian caspases are classified into two major groups: apoptotic and inflammatory caspases (McIlwain et al., 2013; Van Opdenbosch and Lamkanfi, 2019). Apoptotic caspases are further divided into initiator and effector caspases. Initiator caspases include caspase-8 and caspase-9, which mediate the extrinsic and intrinsic apoptosis, respectively (Aral et al., 2019). Caspase-8 is recruited to and activated by the death-inducing signaling complex (DISC) (Dickens et al., 2012; Schleich et al., 2013), while caspase-9 is engaged by the mitochondrial cytochrome c-induced, Apaf-1-organized apoptosome (Bratton and Salvesen, 2010; Rodriguez and Lazebnik, 1999). Both initiator caspases directly process and activate downstream effector caspase-3 and -7, which often leads to apoptotic cell death (Boucher et al., 2011; Kuida, 2000; Stennicke et al., 1998; Twiddy and Cain, 2007).

Caspase-2 is the most conserved but functionally poorly defined member of the caspase family; it is considered an initiator caspase due to its domain similarity with caspase-9. Caspase-2 consists of two domains: an N-terminal caspase activation and recruitment domain (CARD) that responds to upstream signals and a C-terminal caspase protease domain that processes target substrates (Bouchier-Hayes and Green, 2012; Fava et al., 2012). The platform for caspase-2 activation is thought to be a multi-component complex called the PIDDosome, which comprises three proteins, namely PIDD1, RAIDD, and caspase-2 (Ahmad et al., 1997; Tinel and Tschopp, 2004). *PIDD1* is a p53-induced gene and can promote apoptosis (Lin et al., 2000). RAIDD is a two-domain adaptor protein with its N-terminal death domain (DD) interacting with the DD of PIDD1, while its C-terminal CARD engages caspase-2 through homotypic CARD–CARD interaction (Park et al., 2007). A well-known but controversial view is that formation of the PIDDosome is triggered by DNA damage-induced genotoxic stress (Janssens and Tinel, 2012; Tinel and Tschopp, 2004). However, how the PIDDosome senses DNA damage-induced signals and whether there are other unknown signals that can activate the PIDDosome require further investigations.

Several studies report that caspase-2 induces apoptosis by causing mitochondrial cytochrome c release in response to some cellular stresses (Janssens and Tinel, 2012; Paroni et al., 2002; Tu et al., 2006; Upton et al., 2008). Caspase-2 is also suggested to cleave pro-apoptotic Bcl2 family protein BID (Bonzon et al., 2006; Upton et al., 2008; Wagner et al., 2004), presumably to activate the mitochondrial apoptosis pathway (Brown-Suedel and Bouchier-Hayes, 2020). While this mechanism is yet to be fully validated, other studies propose that caspase-2 induces mitochondrial outer membrane permeabilization, which is independent of BID and other intracellular factors (Bonzon et al., 2006; Enoksson et al., 2004; Guo et al., 2002). Thus, the exact mechanisms of caspase-2 activation and caspase-2-induced apoptosis remain inconclusive and even controversial.

To date, few physiological contexts are linked to caspase-2-mediated apoptosis. Unlike caspase-8/9 whose knockout is embryonic lethal (Kuida et al., 1998; Varfolomeev et al., 1998; Zheng et al., 1999), *Casp2*^−/−^ mice are born at expected Mendelian frequencies and develop normally (Bergeron et al., 1998; O’Reilly et al., 2002), indicating its dispensable role in embryonic development. Interestingly, mutations in *RAIDD* or *PIDD1* that impair caspase-2 activation cause neurodevelopmental disorders with pachygyria and psychiatric features (Di Donato et al., 2016; Sheikh et al., 2021; Zaki et al., 2021). Biallelic truncating variants in *CASP2* cause similar neurodevelopmental disorder with lissencephaly (Uctepe et al., 2024), suggesting that the PIDD1–RAIDD-caspase-2 axis is crucial for normal gyration of developing human neocortex as well as cognition and behavior. Moreover, although *Casp2*^−/−^ mice do not develop tumors spontaneously (Shalini et al., 2012), loss of *Casp2* promotes tumorigenesis in many mouse models including Eμ-*Myc* lymphoma (Ho et al., 2009), *c-Neu*-driven mammary carcinoma (Parsons et al., 2013), and *ATM*^−/−^ lymphoma (Puccini et al., 2013). Thus, caspase-2 may function as a potential tumor suppressor. Such function may not necessarily be directly linked to PIDD1 as loss of *PIDD1* instead delays *Myc*-driven lymphomagenesis (Manzl et al., 2012).

In this study, we identified BID as the functional substrate of caspase-2 through an unbiased genetic screen. Cleavage of BID by caspase-2 activates the mitochondria-mediated intrinsic pathway, which determines PIDDosome-induced apoptosis. In addition, we discovered a caspase-2 agonist, HUHS015, through a chemical screen and improved its potency through chemical modifications. We further resolved the mechanism of HUHS015 action to be independent of PIDDosome but through directly targeting the interdomain linker in caspase-2. Two-residue differences there allow HUHS015 to discriminate between human and mouse caspase-2 and activate the former specifically. The HUHS015-series of caspase-2 activators may have great potential for developing new treatments for caspase-2-related neurodevelopmental disorders or cancers.

## Results

### Caspase-2 and RAIDD but not PIDD1 are widely and abundantly expressed

During our study of the PIDDosome pathway, we observed that endogenous expression of RAIDD and caspase-2 was readily detected in commonly used cell lines such as HeLa, Jurkat, and U937 cells, which is consistent with the RNA-seq data in the public domain. However, these cells expressed little or extremely low levels of PIDD1. To further investigate this, we profiled a panel of 60 different human cancer cell lines (NCI-60) by immunoblotting and found that 22 of them expressed RAIDD and caspase-2 at a level equal to or greater than that in HeLa cells (Fig. S1A). Due to the unavailability of a sensitive antibody capable of detecting endogenous PIDD1, we performed quantitative reverse transcription polymerase chain reaction (qRT-PCR) analyses and found that the mRNA level of *PIDD1* was consistently low across all 22 selected cells, as indicated by a mean Ct value of approximately 30 (Fig. S1B).

### PIDDosome activates caspase-2 and induces apoptosis in Jurkat and HL-60 cells

The above data suggests that *PIDD1* expression is probably transcriptionally induced by an unknown signal for being ready to activate RAIDD and caspase-2. Indeed, PIDD1 is known to be a p53-induced protein (Lin et al., 2000). Alternatively, *PIDD1* may not function together with RAIDD and caspase-2 as a *bona fide* cell death signaling axis. Our biochemical analyses suggested that the purified DD of PIDD1 (PIDD1-DD), full-length RAIDD protein, and the CARD domain of caspase-2 (caspase-2-CARD) formed a stable ternary complex when eluted from a gel-filtration column (Fig. S1C). While the ternary complex assembly is in line with the PIDDosome concept proposed in previous studies, there has been no reported data suggesting that PIDD1-DD is sufficient to induce caspase-2-mediated cell death via RAIDD. We also failed to observe evident cell death when PIDD1-DD was ectopically expressed in the RAIDD and caspase-2-positive cell lines.

**Figure 1.**
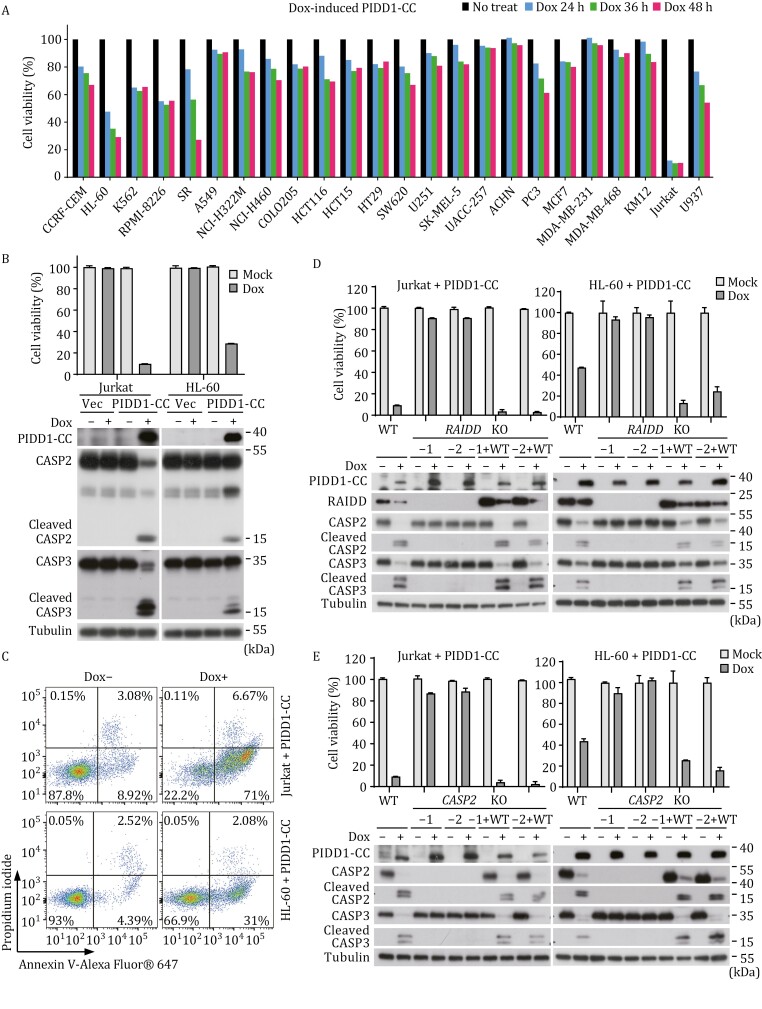
**PIDDosome-mediated caspase-2 activation induces apoptosis in various cancer cell lines.** (A) Doxycycline (Dox)-induced expression of PIDD1-CC triggers cell death in multiple *RAIDD/CASP2* double-positive cancer cell lines. ATP-based cell viability measured at the indicated timepoint is shown. (B and C) Inducible expression of PIDD1-CC causes cell death and caspase-2 and -3 activation in Jurkat and HL-60 cells. Jurkat and HL-60 cells were treated with dox for 5 h and 9 h, respectively. (B) ATP-based cell viability and immunoblotting detecting caspase-2 and -3 processing in Jurkat and HL-60 cells are shown. (C) Representative flow cytometry pseudo-color dotplots of propidium iodide and annexin V-stained Jurkat and HL-60 cells are shown. (D and E) RAIDD and CASP2 are both essential for PIDD1-CC-induced caspase-2 and -3 activation and cell death in Jurkat and HL-60 cells. Two *RAIDD*^−/−^ (KO) clones (D) and two *CASP2*^−/−^ (KO) clones (E) of Jurkat and HL-60 cells, as well as the knockout clones complemented with wild-type (WT) RAIDD and caspase-2, respectively, were stimulated by dox-induced expression of PIDD1-CC. ATP-based cell viability and immunoblotting detecting caspase-2 and -3 processing are shown. ATP-based cell viability in (B, D, and E) is expressed as mean ± s.d. from three technical replicates. All data are representative of three independent experiments.

Full-length PIDD1 (910 residues) contains an N-terminal LRR domain and a C-terminal DD, between which are tandem ZU5 domains followed by a UPA domain (Fig. S1D). The ZU5-UPA module has an intrinsic autoprocessing, proteolytic activity; indeed, PIDD1 is known to be auto-cleaved sequentially at two sites, S446 between the two ZU5 domains and S588 between the second ZU5 domain and the UPA domain (Fig. S1D) (Tinel et al., 2007). The two cleavages generate a PIDD1-C (residues 447–910) and a PIDD1-CC (residues 589–910) fragment, respectively. We further found that doxycycline-induced overexpression of PIDD1-CC, but not PIDD1-C, caused cell death to various extents in Jurkat, U937 and the 22 cancer cell lines, according to measurements of the ATP level in the cells (Fig. 1A). Among these cells, Jurkat and HL-60 cells showed the most robust cell death (Fig. 1A and 1B), in which processed, active forms of caspase-2 and caspase-3 were readily detected (Fig. 1B). Flow cytometry showed that the dying cells became Annexin V-positive but PI-negative, characteristic of apoptotic death; the percentage of Annexin V^+^/PI^−^ population reached more than 70% in Jurkat cells (Fig. 1C). This data for the first time demonstrates directly that PIDDosome can activate caspase-2, causing apoptosis. The data also strongly suggests that activation of RAIDD/caspase-2 requires auto-cleavage of PIDD1 at S588 to generate PIDD1-CC despite that the shorter PIDD1-DD alone could readily bind RAIDD and form a ternary complex with caspase-2.

We further showed that knockout of *RAIDD* in Jurkat cells abolished PIDDosome-stimulated apoptosis as well as the processing of caspase-2 and caspase-3, both of which were restored by re-expression of exogenous RAIDD (Fig. 1D). The same results were obtained in HL-60 cells (Fig. 1D). We additionally generated *CASP2*^−/−^ Jurkat and HL-60 cells, in which induced expression of PIDD1-CC could no longer trigger apoptosis (Fig. 1E). Moreover, the cleavage and activation of caspase-3 were diminished in the *CASP2*^−/−^ cells (Fig. 1E). Complementing the *CASP2*^−/−^ cells with a caspase-2-expressing plasmid restored PIDD1-CC-induced apoptosis as well as the cleavage of caspase-3 (Fig. 1E). Thus, activation of caspase-2 is critically required for PIDD1-CC-stimulated apoptosis, which is upstream of caspase-3 activation.

### A genome-wide CRISPR/Cas9 screen identifies BID being required for PIDDosome-induced caspase-2-dependent apoptosis

To investigate the signaling mechanism underlying caspase-2-mediated apoptosis stimulated by the PIDDosome, we endeavored to develop a fluorescence-activated cell sorting (FACS)-based genome-wide CRISPR-Cas9 screen in cells responsive to PIDD1-CC overexpression. Although Jurkat and HL-60 cells are competent in PIDD1-CC-stimulated caspase-2 activation and apoptosis, both cells are technically challenging for a genome-wide genetic screen. We turned to the U937 cells and first examined whether this cell line features the PIDDosome-caspase-2 axis for apoptosis induction, like in Jurkat and HL-60 cells. The doxycycline-induced PIDD1-CC linked with mRuby3 (RFP) through a 2A self-cleaving sequence (T2A peptide) was introduced into the U937 cells, which produced isolated RFP from T2A self-cleavage. The RFP signal was used for sorting PIDD1-CC-positive cells in the FACS-based screen. ZsGreen (GFP) was additionally expressed in these cells to discriminate live cells from apoptotic ones. Upon doxycycline treatment, the PIDD1-CC-expressing U937 cells (a selected single clone) underwent evident caspase-2 processing and apoptosis, both of which were blocked by knockout of *RAIDD* or *CASP2* (Fig. S2A and S2B). Complementing the *RAIDD*^−/−^ or *CASP2*^−/−^ cells with exogenously expressed RAIDD or caspase-2, respectively, restored PIDD1-CC-induced caspase-2 processing and apoptosis (Fig. S2A and S2B).

**Figure 2.**
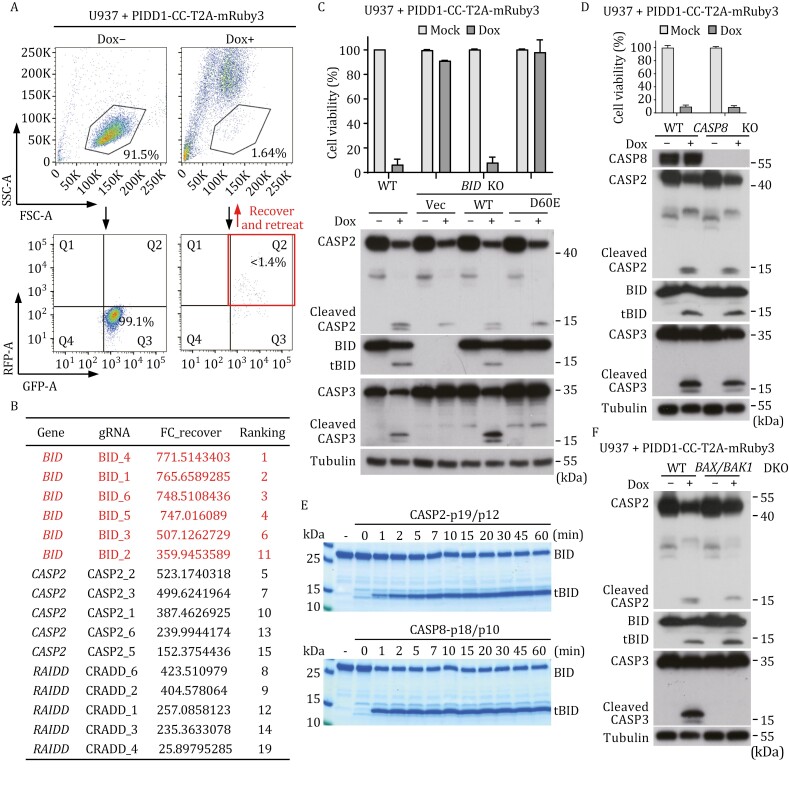
**Unbiased genetic screen identifies that BID is required for PIDDosome-induced apoptosis through cleavage by caspase-2.** (A) FACS-based genome-wide CRISPR-Cas9 screen in U937 cells with inducible expression of PIDD1-CC-T2A-mRuby3. The U937 cells were treated with dox for 12 h before FACS sorting. The surviving cells were recovered and retreated with dox until three rounds of enrichment. Representative flow cytometry pseudo-color dotplots for the first round of FACS are shown. (B) Top gRNA hits enriched from the genome-wide genetic screen of PIDDosome-induced cell death in U937 cells. (C) BID is required for PIDD1-CC-induced caspase-2-dependent cell death in U937 cells. WT and *BID*^−/−^ (KO) U937 cells, as well as the knockout U937 cells rescued with WT or D60E mutant of BID, were stimulated by dox-induced expression of PIDD1-CC. ATP-based cell viability and immunoblotting detecting BID, caspase-2 and -3 processing are shown. (D) Validation of the role of caspase-8 in PIDD1-CC-induced cleavage of BID and cell death. WT and *CASP8*^−/−^ (KO) U937 cells were stimulated by dox-induced expression of PIDD1-CC. ATP-based cell viability and immunoblotting detecting BID, caspase-2 and -3 processing are shown. (E) *In vitro* cleavage of BID by active form of caspase-2 and -8. Recombinant BID proteins were incubated with active caspase-2 or caspase-8 for indicated time and the samples were subjected to SDS-PAGE analyses. (F) Validation of the roles of BAX and BAK in PIDD1-CC-induced BID and caspase-3 processing by immunoblotting. WT and *BAX*^−/−^*BAK*^−/−^ (DKO) U937 cells were stimulated by dox-induced expression of PIDD1-CC. In panels (C, D, and F), the cells were treated with dox for 12 h before cell viability measurement and immunoblotting analyses. ATP-based cell viability in panels (C and D) is expressed as mean ± s.d. from three technical replicates. Data in panels (C, D, E, and F) are representative of three independent experiments.

For the screen, a library of knockout U937 cells harboring PIDD1-CC-T2A-mRuby3 and GFP were treated with doxycycline to induce PIDDosome-stimulated apoptosis (Fig. 2A). The pool of RFP/GFP-double-positive cells, obtained after three cycles of doxycycline treatment and FACS sorting, were subjected to gRNA sequencing to identify enriched clones. Among the top 2,000 gRNA hits, nine genes were hit by four or more gRNAs; except for *RAIDD*, *CASP2*, and a known apoptosis gene *BID*, the other six genes did not pass subsequent validation using their knockout cell lines (Fig. S3). Notably, *BID* was targeted by all the 6 gRNAs with five of them on the top of the ranking list (Fig. 2B). *BID* encodes a BH3-only proapoptotic protein, belonging to the BCL-2 family. In PIDD1-CC-stimulated WT U937 cells, BID was readily processed into a specific truncated form, concurrent with caspase-2 and caspase-3 activation and occurrence of apoptosis (Fig. 2C). Importantly, *BID*^−/−^ in the U937 cells did not affect the processing of caspase-2 but abolished caspase-3 activation and apoptosis (Fig. 2C). Re-expression of exogenous BID in the *BID*^−/−^ cells restored caspase-3 processing as well as the cell death (Fig. 2C). Thus, BID likely functions downstream of PIDDosome-activated caspase-2, which is required for caspase-3 activation and subsequent apoptosis. It is worth noting that BID was widely expressed in the 22 cancer cell lines (Fig. S2C). The differential death responses in these cells to PIDD1-CC expression did not correlate with BID expression, suggesting a regulation of PIDD1-CC-induced apoptosis by other known or unknown factors.

### PIDDosome-activated caspase-2 cleaves BID to activate intrinsic mitochondrial apoptosis pathway

Previous studies have shown that extrinsic apoptotic stimuli sensed by death receptors induce DISC in which caspase-8 is activated by autoprocessing and further processes caspase-3/7 to induce apoptosis (Boucher et al., 2011; Stennicke et al., 1998). Active caspase-8 cleaves BID at D60 (Li et al., 1998); the truncated, active form of BID (tBID) removes the inhibition of antiapoptotic BCL-2 family proteins on BAX and BAK. Unleashed BAX/BAK oligomerize together in the mitochondrial outer membrane (MOM) and permeabilize the MOM to release cytochrome c into the cytosol, initiating Apaf-1 and caspase-9-dependent intrinsic pathway of apoptosis (Rodriguez and Lazebnik, 1999). We observed that complementing the *BID*^−/−^ cells with a BID D60E mutant completely diminished its processing, in which caspase-3 activation and cell death were both suppressed (Fig. 2C). This suggests that PIDDosome-induced caspase-2 activation leads to a functional cleavage of BID at D60 which governs subsequent caspase-3 activation and apoptosis. Knockout of *CASP8* in the U937 cells had little effects on BID processing as well as caspase-2 and caspase-3 activation, and the stimulated *CASP8*^−/−^ cells showed robust cell death comparable with that in WT U937 cells (Fig. 2D). Thus, BID is not processed by caspase-8 during PIDDosome-stimulated apoptosis.

We investigated whether PIDDosome-activated caspase-2 directly cleaves BID. Purified active caspase-2 was found capable of processing BID *in vitro*, which was as efficient as the cleavage by caspase-8 and generated the same-size truncated BID (Fig. 2E). This result clarifies that caspase-2 could functionally process BID into tBID (Bonzon et al., 2006; Upton et al., 2008; Wagner et al., 2004). Meanwhile, unlike caspase-8, which could robustly process full-length caspase-3 and -7 (the enzymatically deficient C163A and C186A mutant, respectively), caspase-2 could not cleave caspase-3/7 *in vitro* (Fig. S2D and S2E). This agrees with the notion that caspase-2 induces caspase-3 activation and apoptosis through BID in PIDDosome-stimulated cells (Fig. 2C). Consistently, knockout of *BAX* and *BAK* in the PIDDosome-stimulated U937 cells blocked caspase-3 activation without affecting caspase-2 autoprocessing and the cleavage of BID (Fig. 2F). In contrast, the *BAX*^−/−^/*BAK*^−/−^ U937 cells showed intact caspase-8 cleavage of BID and caspase-3 activation upon TNFα plus cycloheximide (CHX) treatment (Fig. S2F). Similarly, *BAX*^−/−^/*BAK*^−/−^ HeLa cells were absent from caspase-3 activation in response to PIDDosome stimulation while TNFα plus CHX treatment bypassed the requirement of the mitochondrial pathway to induce caspase-3 activation (Fig. S2G). In both contexts, BID was efficiently processed into tBID (Fig. S2G).

### High-throughput screen identifies a compound that can specifically trigger caspase-2-mediated apoptosis

**Figure 3.**
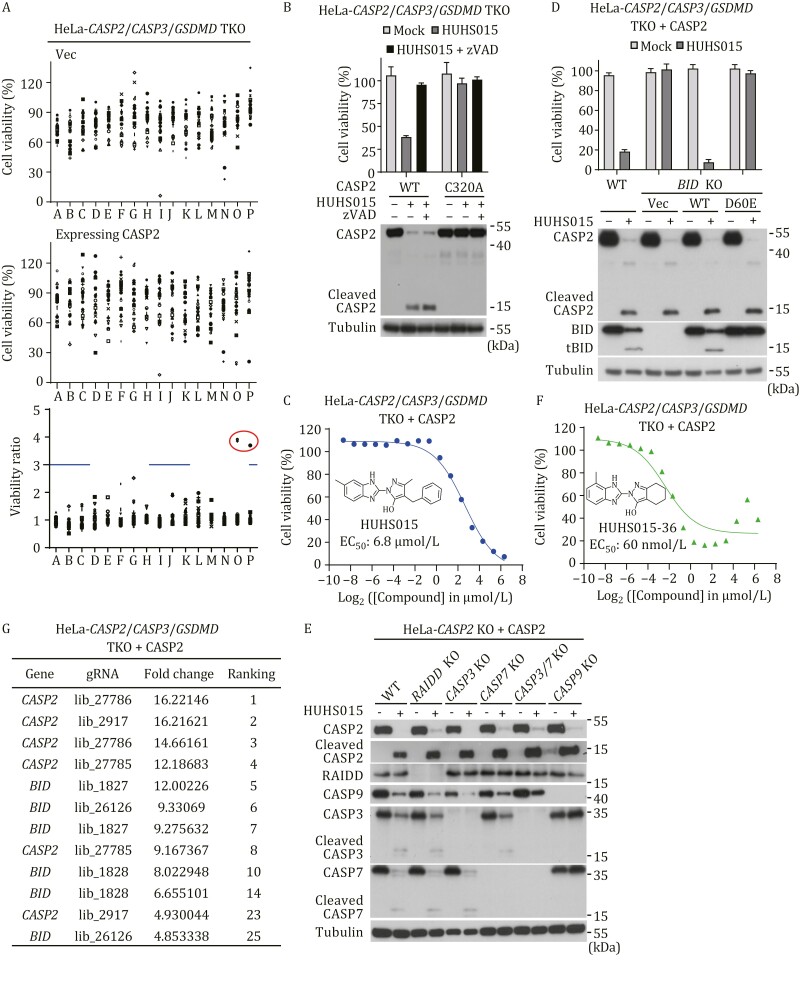
**Chemical screen identifies HUHS015 as an agonist of human caspase-2.** (A) Screen raw data and selectivity analyses of the compound plate containing HUHS015. The ATP-based viability data of both HeLa-*CASP2*^−/−^*CASP3*^−/−^*GSDMD*^−/−^ (TKO) (control group) and TKO cells with exogenous caspase-2 expression (experimental group) are shown. The ratios of the cell viability between the two groups are calculated with 3 as a cutoff for positive compound hit selection. (B) HUHS015-induced cell death depends on caspase-2 activity. HeLa TKO cells expressing WT or enzymatically deficient C320A mutant of caspase-2 were treated with HUHS015. ATP-based cell viability and immunoblotting of caspase-2 cleavage are shown. (C and F) The structures and potencies of HUHS15 (C) and its optimized derivative HUHS15-036 (F). HeLa TKO cells with exogenous caspase-2 expression were treated with titrated compounds. The ATP-based viability data were plotted with compound concentration for EC_50_ calculation. (D) BID is required for HUHS15-induced caspase-2-dependent cell death. WT and *BID*^−/−^ (KO) HeLa TKO cells with exogenous caspase-2 expression, as well as the knockout cells rescued with WT or D60E mutant of BID were treated with HUHS15. ATP-based cell viability and immunoblotting detecting caspase-2 and BID processing are shown. (E) HUHS15-induced caspase-3/7 activation relies on caspase-9 but is independent of PIDDosome. *CASP2*^−/−^ (KO) HeLa cells with exogenous caspase-2 expression in the indicated knockout background were treated with HUHS15. Processing of caspase-2, -3, and -7 was analyzed by immunoblotting. (G) Top gRNA hits from the genetic screen of HUHS015-36-induced apoptosis in HeLa TKO cells with exogenous caspase-2 expression. In all the data, indicated cells were treated with the compound for 5 h (for HUHS015-36) or 9 h (for HUHS015) before cell viability measurement and immunoblotting analyses. ATP-based cell viability in panels (B and D) is expressed as mean ± s.d. from three technical replicates. Data in panels (B–F) are representative of three independent experiments.

Specific chemical compounds are powerful tools for dissecting target-related signaling mechanisms in biological contexts (Beck et al., 2022; Zhang et al., 2012). Prompted by this notion and possibly to identify a compound that could activate the PIDDosome, we designed a cell death-based high-throughput chemical screen in HeLa cells, in which a triple knockout of *CASP2*, *CASP3*, and *GSDMD* were constructed. *CASP3* and *GSDMD* deficiency served as the background to minimize compound-induced nonspecific cytotoxicity and undesired pyroptosis. Using the triple knockout cells as the control, additional expression of exogenous caspase-2 allows the identification of compounds that can specifically activate the caspase-2-BID pathway and trigger apoptosis through caspase-7. By screening a library of 7,217 compounds, a hit named HUHS015 was identified by virtue of its induction of robust cell death selectively in caspase-2-expressing but not the control triple knockout cells (Fig. 3A). In accordance with the apoptosis induction, HUHS015 induced evident processing of caspase-2 expressed in the triple-knockout cells (Fig. 3B). This indicated activation of caspase-2 as the protease-deficient C320A mutant of caspase-2 was not processed upon HUHS015 treatment (Fig. 3B). Accordingly, expression of caspase-2 C320A rendered no cell death in response to HUHS015 treatment (Fig. 3B). Notably, HUHS015-induced caspase-2 processing was not blocked by the pan-caspase inhibitor zVAD (Fig. 3B), consistent with the notion that zVAD is a poor inhibitor of caspase-2 (Poreba et al., 2019). Thus, HUHS015 specifically induces caspase-2 activation and caspase-2-mediated cell death, and its half-maximal effective concentration (EC_50_) was around 6.8 μmol/L (Fig. 3C).

We then explored whether HUHS015-stimulated caspase-2 activation shares the same downstream mechanism with PIDDosome activation to cause cell death. As expected, HUHS015 treatment of caspase-2-expressing triple-knockout HeLa cells induced cleavage of BID, and the cell death was diminished by knockout of *BID* (Fig. 3D). Both the BID cleavage and the cell death were restored by re-expression of WT BID but not its D60E mutant in the *BID*^−/−^ cells (Fig. 3D). Thus, HUHS015-stimulated caspase-2 activation, as that induced by PIDDosome, triggers apoptosis through the BID-dependent mitochondrial pathway. In line with this realization, HUHS015 induced robust processing of caspase-3 and -7, both of which were abolished when *CASP9* was deleted from the cells (Fig. 3E). Knockout of *CASP3* or *CASP7* alone did not block HUHS015-induced activation of caspase-2 (Fig. 3E) due to their redundant functions. This also well explains why our chemical screen could be achieved in the *CASP3*^−/−^ cells.

The resemblance of downstream signaling between PIDDosome and HUHS015-induced caspase-2 activation prompted us to investigate whether HUHS015 has any connection with PIDDosome signaling. When *RAIDD* was knocked out from the caspase-2-expressing HeLa cells, HUHS015 could still induce caspase-2 processing as well as caspase-3/7 activation, comparable to that in the *RAIDD*-sufficient cells (Fig. 3E). Thus, HUHS015-induced caspase-2 activation is independent of the PIDDosome or at least RAIDD in the PIDDosome.

### Structure–activity relationship analyses of HUHS015 and its derivatives

To obtain an optimized, more potent derivative of HUHS015, we analyzed its structure–activity relationship (SAR). The core structure of HUHS015 features two parts, a benzimidazole directly linked to a hydroxylpyrazole, both of which bear a methyl substitution in the ring. The hydroxylpyrazole also contains a benzyl group at the ortho-position of the hydroxyl group (Figs. 3C and S4A). We first sought commercially available analogs of HUHS015 and examined whether some of them could recapitulate the cell death-inducing activity of HUHS015. Among the 34 analogs tested, analog 15 (A15) showed the highest potency with an EC_50_ of about 0.94 μmol/L (Fig. S4A and S4G). The structure of A15 indicates that a cyclohexane fusion with the hydroxylpyrazole instead of a phenyl group substitution could improve the potency. The activities of other analogs, such as A1 and A2, also provide valuable insights into the SAR, which indicates that the methyl group in the benzimidazole ring is not necessary but the hydroxyl group in the hydroxylpyrazole ring is indispensable for the activity (Fig. S4A).

We used the core structure of A15 as the starting point for subsequent SAR analyses. We generated a series of derivatives of A15 by individually changing the fused ring on the hydroxylpyrazole and the benzimidazole ring through ring-structure diversification, substitutions in the ring, or heteroatoms incorporation. For the saturated cycloalkane fusion with the hydroxylpyrazole, found that six, seven, and eight-atom rings are equivalent. Small alkyl group substitution on the cyclohexane maintained the activity, but heteroatoms incorporation into the cyclohexane largely impaired the compound potency (Fig. S4B). The SAR was narrow for the benzimidazole, and any changes in the structure of imidazole ring resulted in loss of activity (Fig. S4C). The phenyl group fused with the imidazole ring is absolutely required as compound 32 (with no phenyl group) lost the activity (Fig. S4C). The direct linkage between the benzimidazole and the hydroxylpyrazole is also crucial because a single methylene insertion in the linker killed the cell death-inducing activity (Fig. S4C). For the phenyl group in the benzimidazole part, introducing a methyl group at the R4 position gave marked improvement in the compound potency, and the EC_50_ of compound 36 reached about 60 nmol/L (Figs. 3F, S4D and S4G). A nitrogen atom incorporation into the X position of the phenyl group could be tolerated, and the resulting compound 53 also showed a high potency with an EC_50_ of about 120 nmol/L (Fig. S4D and S4G). By fixing the benzimidazole part with a methyl group at the R4 position, we further explored whether there is a space to optimize the fused ring on the hydroxylpyrazole. Like SAR analyses on the A15 background, cycloheptane fusion showed comparable potency as observed with compound 36; the resulting compound 59 had an EC_50_ of ~80 nmol/L (Fig. S4E and S4G). As for the cycloheptane-fused hydroxylpyrazole, we did not obtain more potent compounds by re-altering the structures of the phenyl group in the benzimidazole part (Fig. S4F). Taking all these analyses together, we obtained derivatives with largely improved potency based on the initial hit HUHS015.

### HUHS015-derived compounds directly target caspase-2 for activation

We used the best compound HUHS015-36 as a tool to further investigate the mechanism of compound action in caspase-2 activation and cell death induction. To identify potential component(s) targeted by the compound or in mediating the compound action, we again performed an unbiased genome-wide genetic screen using HUHS015-36 as a cell death trigger. For the screen, the caspase-2-expressing triple knockout HeLa cells were subjected to CRISPR-Cas9-mediated random mutagenesis, and the pool of mutant cells was then treated with HUHS015-36. Over 95% of the treated cells underwent apoptosis, and the surviving cells were collected for gRNA sequencing. The top gRNA enriched mainly hit *CASP2* and *BID*, encoding two key components already validated when analyzing the compound-induced cell death (Fig. 3G). As expected, the PIDDosome components PIDD1 and RAIDD were not hit in the nearly exhaustive genetic screen. We further performed targeted knockout of *PIDD1* and *RAIDD*, which confirmed that neither *PIDD1* nor *RAIDD* deficiency had any effects on HUHS015-36-induced caspase-2 activation and cell death (Fig. S5A). Importantly, no other genes with reliable enrichments of multiple gRNA were selected out of the results of the CRISPR-Cas9 screen.

**Figure 4.**
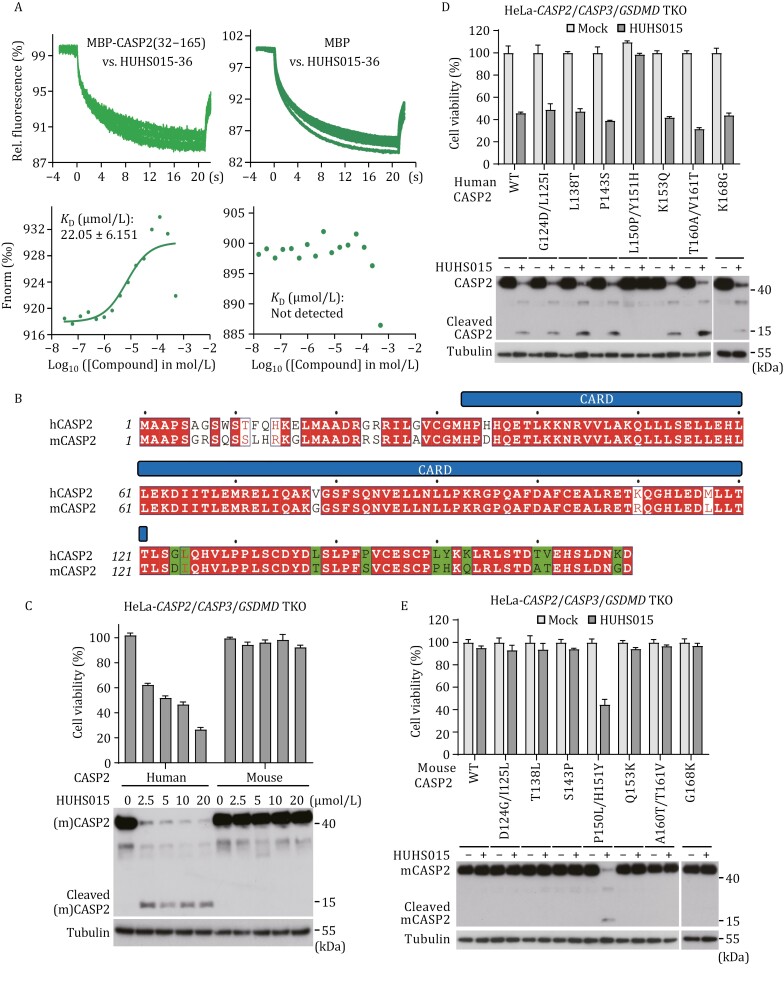
**HUHS015 activates human caspase-2 directly through specific targeting of the interdomain linker of caspase-2.** (A) The direct binding between HUHS015-36 and MBP-fused human caspase-2 pro-domain was detected by microscale thermophoresis (MST). MST profiles for raw binding signals and affinity (*K*_D_) measurement are shown. The calculated dissociation constant (*K*_D_) is expressed as means ± s.d. from two determinations. (B) Sequence alignment of the pro-domain (residues 1–169) of human and mouse caspase-2. CARD domain annotation is shown on top of the sequences. Identical residues are in red background and conserved ones are in red. Numbers of starting residues are indicated on the left. Different residues in the interdomain linker (residues 122–169) were highlighted with green color. (C) HUHS015 activates human but not mouse caspase-2. HeLa TKO cells rescued with human or mouse caspase-2 expression were treated with HUHS015 for the indicated time. ATP-based cell viability and immunoblotting detecting caspase-2 processing are shown. (D and E) Profiling the crucial sites determining the differential responsiveness of human and mouse caspase-2 to HUHS015 activation. HeLa TKO cells rescued with WT human caspase-2 and indicated mutants (D) or WT mouse caspase-2 and indicated mutants (E) were treated with HUHS015 for 5 h. ATP-based cell viability and immunoblotting detecting caspase-2 processing are shown. ATP-based cell viability in panels (C–E) is expressed as mean ± s.d. from three technical replicates. Data in panels (C–E) are representative of three independent experiments.

The above results prompted us to reason whether HUHS015-36 could directly target caspase-2 for activation without the requirement of any other mediator. To directly test this hypothesis, we tried to obtain purified caspase-2 protein. The preparation of full-length caspase-2 protein is known to be technically challenging. Meanwhile, the CARD domain of a caspase often plays the role in sensing upstream signals, as exemplified by the CARD of caspase-2 that is recruited by RAIDD through the CARD–CARD interaction for assembling the PIDDosome. Following extensive attempts of protein expression and purification, we succeeded in obtaining maltose-binding protein (MBP)-fused caspase-2 pro-domain comprising the typical CARD domain (residues 32–121) and the following linker region (residues 122–165). Notably, MicroScale Thermophoresis (MST) measurements detected a reliable binding between HUHS015-36 and MBP-caspase-2^pro-domain^, but not between HUHS015-36 and MBP alone (Fig. 4A). The binding constant (*K*_D_) was determined to be ~22 μmol/L (Fig. 4A). This data strongly suggest that HUHS015-36 can directly act on caspase-2 and that the HUHS015 series of compounds are efficient small-molecule agonists of caspase-2.

### HUHS015-like agonists distinguish human caspase-2 from its mouse ortholog

Mouse caspase-2 shares remarkable sequence homology with human caspase-2, especially in the pro-domain where sequence similarity reaches ~88% (Fig. 4B). The CARD domain of mouse caspase-2 only differs from its human counterpart by four amino acids (Fig. 4B). Consistently, human and mouse caspase-2, when expressed separately in *CASP2*^−/−^ HeLa cells, exhibited similar processing upon PIDDosome activation by doxycycline-induced PIDD1-CC, and the induced death responses were also comparable (Fig. S5B). When human caspase-2-expressing HeLa cells were treated with a series of titrating doses of HUHS015, the percentage of cell death increased accordingly, accompanied by evident caspase-2 processing (Fig. 4C). In a striking contrast, mouse caspase-2-expressing HeLa cells showed no apoptosis even when treated with high concentrations of HUHS015, with no caspase-2 processing detected as well (Fig. 4C). This suggests that HUHS015 can distinguish human and mouse caspase-2.

The contrasting responses of the two caspases to HUHS015 might result from sequence differences in the interdomain linker in the pro-domain. Sequence alignment revealed ten-residue differences in the interdomain linker (Fig. 4B). Exchanging these residues individually or jointly for consecutive ones between human and mouse caspase-2 showed that most of the substitutions did not alter the sensitivity to activation by HUHS015 (Fig. 4D and 4E). However, replacing L150 and Y151 in human caspase-2 with mouse P150 and H151, respectively, abolished HUHS015-induced caspase-2 activation as well as the cell death response (Fig. 4D). Conversely, substitution of P150 and H151 in mouse caspase-2 with L150 and Y151, respectively, rendered mouse caspase-2 capable of being activated by HUHS015 and thereby inducing apoptosis (Fig. 4E). The importance of L150/Y151 for human caspase-2 activation by HUHS015 indicates these residues or the associated structure are likely involved in binding to the compound agonist. Supporting this notion, MST measurement failed to detect confident binding between HUHS015-36 and the L150P/Y151H mutant of MBP-tagged human caspase-2^pro-domain^ (Fig. S5C).

**Figure 5.**
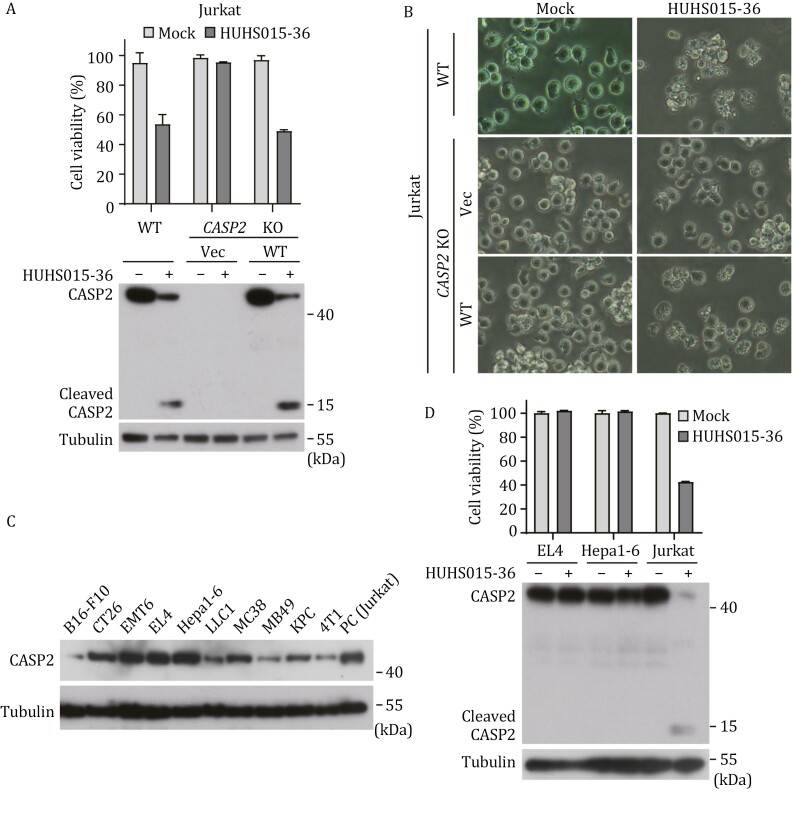
**HUHS015-36 activates endogenous caspase-2 to induce apoptosis in Jurkat cells but not mouse cells**. (A and B) HUHS015-36 activates endogenous caspase-2 and induces apoptosis in human Jurkat cells. WT and *CASP2*^−/−^ (KO) Jurkat cells, as well as *CASP2*^−/−^ cells rescued with WT caspase-2 were treated with HUHS015-36 for 8 h. ATP-based cell viability and immunoblotting detecting caspase-2 cleavage are shown in (A). Representative images were shown in (B). (C) Profiling the expression of endogenous caspase-2 in mouse cells. Lysates of 10 mouse cancer cell lines were analyzed by immunoblotting using indicated antibodies. Human Jurkat cells were included as a positive control. (D) HUHS015-36 cannot activate mouse caspase-2. Mouse cancer cell lines EL4 and Hepa1-6, as well as human Jurkat cells, were treated with or without HUHS015-36 for 8 h. ATP-based cell viability and immunoblotting detecting caspase-2 cleavage are shown. ATP cell viability (A and D) is expressed as mean ± s.d. from three technical replicates. All data are representative of three independent experiments.

We further examined whether HUHS015-derived compounds could activate endogenous caspase-2 in human cells. In WT Jurkat cells, HUHS015-36 treatment induced evident caspase-2 processing and cell death, both completely diminished by *CASP2* knockout (Fig. 5A and 5B). Complementing the *CASP2*^−/−^ Jurkat cells with exogenous caspase-2 restored HUHS015-36-induced apoptosis, in which caspase-2 was expectedly processed (Fig. 5A and 5B). We also profiled caspase-2 expression in a panel of 10 mouse tumor cell lines and observed highly variable expression levels (Fig. 5C). In the commonly used mouse tumor cells, like CT26 (colorectal carcinoma) and MC38 (colon adenocarcinoma), endogenous caspase-2 expression was modest, comparable to that in human Jurkat cells (Fig. 5C). In B16-F10 (melanoma), LLC1 (Lewis lung carcinoma), MB49 (bladder carcinoma), KPC (pancreatic ductal adenocarcinoma), and 4T1 (mammary carcinoma) cells, caspase-2 was expressed at much lower levels (Fig. 5C). In contrast, EMT6 (mammary carcinoma), EL4 (T lymphoblast), and Hepa1-6 (hepatoma) feature robust caspase-2 expression, much higher than in Jurkat cells (Fig. 5C). When EL4 and Hepa1-6 cells were treated with HUHS015-36, no caspase-2 processing and cell death were detected (Fig. 5D). These data corroborate that HUHS015-derived compounds are effective agonists for activating endogenous caspase-2 to induce apoptosis in human but not in mouse cells.

## Discussion

Since its identification as an initiator caspase, caspase-2 has been extensively studied and functionally linked to apoptosis through its activation by cellular stresses, including DNA damage, mitotic catastrophe, ER stress, and metabolic imbalance. Although the biological implications of caspase-2 seem to become clearer, the precise mechanisms for its activation remain elusive. It has been speculated that rapid overexpression of PIDD1, as a p53-induced protein, may cause its activation through self-aggregation (Lin et al., 2000). Also, centrosome overduplication caused by cytokinesis disruption can recruit PIDD1 to stimulate PIDDosome formation and caspase-2 activation (Burigotto et al., 2021; Fava et al., 2017). Among all proposed upstream triggers of caspase-2 activation, the DNA damage-induced PIDDosome signaling complex is the best known to act as an apoptosome-like molecular platform that recruits caspase-2 for proximity-induced autoprocessing. However, whether and how PIDDosome-stimulated caspase-2 activation triggers cell death remains to be clarified and defined.

In this study, we confirmed PIDDosome-induced caspase-2-mediated cell death. The cleavage of BID by caspase-2 has been suggested in previous studies, but its functional relevance with caspase-2 activation, especially its essential role in PIDDosome-induced death signaling, is poorly understood. Through a cell death-based unbiased genetic screen, we identified BID as a physiological substrate of caspase-2 and a molecular determinant for eliciting mitochondria-dependent intrinsic apoptosis in response to PIDDosome activation. The proapoptotic activity of BID is counteracted by other antiapoptotic BCL2 family of proteins. Therefore, the different sensitivities to PIDDosome-induced cell death in various cells may be caused by the distinct expression levels of those antiapoptotic proteins. Our studies highlight the dominant role of BID in determining the apoptotic outcome of the cells. This differs from the well-established caspase-8 cleavage of BID that is auxiliary to (caspase-8) direct activation of caspase-3/7 in inducing cell death.

Our study was initially aimed to answer how DNA damage induces PIDDosome and caspase-2 activation. Unfortunately, we found that caspase-2 activation in HeLa cells caused by DNA damage drugs such as doxorubicin or taxol did not require PIDD1 (data not shown). We then turned to identify and develop a specific chemical tool for investigating the mechanisms underlying PIDDosome activation that functions upstream of caspase-2-mediated cell death. Unexpectedly, we screened out a *bona fide* agonist that can directly activate caspase-2 to induce cell death. Through SAR optimization of the initial hit HUHS015, we generated a few superior derivatives that enable the compound utilization more efficiently. Interestingly, we discovered that two residues of sequence difference in the interdomain linker determine that the HUHS015 series of compounds can only activate human caspase-2 but not mouse homolog. This finding breaks the dogmatic knowledge that the N-terminal CARD domain is used by an initiator caspase to sense the activation stimuli. Further structural studies on the compound-engaged caspase-2 can provide more valuable insights into the binding and agonizing mechanism for these compounds acting on caspase-2, which can fuel more discovery and optimization of caspase-2 agonists.

We also found that caspase-2 is widely expressed in dozens of human cancer cells, which makes activation of caspase-2-mediated cell death a potential strategy to kill cancer. The HUHS015 series of compounds are promising tools for exploring this direction. However, since mouse caspase-2 expressed in the tumor cells cannot be activated by the HUHS015 series of compounds, humanized mice with agonist-competent caspase-2 need to be generated. With the agonist as a useful trigger, the physiological and pathological roles of caspase-2 in different biological contexts could be investigated in the caspase-2-humanized mice.

## Materials and methods

### Plasmids

cDNAs encoding human *CASP2*, *BID*, *RAIDD*, *CASP8*, *PIDD1*, and mouse *Casp2* were synthesized by our in-house gene synthesis facility. For stable expression of indicated genes, cDNAs for human *CASP2*, *BID*, *RAIDD*, *CASP8*, and mouse *Casp2* were inserted into modified FUIMW or FUIGW-Flag vectors. For Tet-On inducible expression in mammalian cells, cDNAs encoding the PIDD1-CC-Flag or PIDD1-CC-T2A-mRuby3 were ligated into pLVX-Tet3GS vectors. The pSpCas9(BB)-2A-GFP plasmid (PX458) (Plasmid #48138), lentiCas9-Blast plasmid (Plasmid #52962), and lentiGuide-Puro plasmid (Plasmid #52963), obtained from Addgene Inc, were used to generate knockout cells. For recombinant expression of PIDDosome complex in *Escherichia coli*, the DNA fragments encoding PIDD1-DD (residues 778-837) and RAIDD were constructed into the pACYCDuet-1 vector with the PIDD1-DD fused with a N-terminal 6× His tag, and DNA for caspase-2-CARD (residues 32–121) was inserted into the pET21a vector with a C-terminal 6× His tag. cDNAs encoding BID and caspase-2 pro-domain (residues 32–165) were constructed into modified pET vectors with an N-terminal 6× His-SUMO tag and an N-terminal 6× His-MBP tag, respectively. To obtain the active forms of caspase-2 and caspase-8 proteins, cDNAs encoding the protease domain (p30) of caspase-2 or -8 were constructed into the pET21a vector with a C-terminal 6× His tag. For recombinant expression of catalytically deficient caspase-3 C163A and caspase-7 C186A mutant proteins, cDNAs encoding *CASP3* and *CASP7* bearing the cysteine mutation were cloned into the pET21a vectors with a C-terminal 6× His tag.

All truncations, deletions, and point mutations were generated by the standard polymerase chain reaction (PCR) cloning method. All plasmids were verified by DNA sequencing.

### Antibodies, compounds, and reagents

Antibodies against caspase-2 (ab179520) and RAIDD (ab76465) were obtained from Abcam. Antibodies against caspase-7 (#9492), cleaved caspase-8 (#9496), caspase-9 (#9508), Bid (#2002), and caspase-3 (#9662) were from Cell Signaling Technology. Anti-Flag (F3165/M2) and anti-tubulin (T5168) antibodies were obtained from Sigma-Aldrich. For Western blot, horseradish peroxidase (HRP)-conjugated anti-mouse IgG (NA931) and HRP-conjugated anti-rabbit IgG (NA934) were purchased from GE Healthcare.

Cycloheximide (C7698) was purchased from Sigma-Aldrich. The pan-caspase inhibitor zVAD (HY-16658) was obtained from MedChemExpress. Human TNF-α (rcyc-htnfa) was from InvivoGen. HUHS015 and its derivatives were synthesized by our in-house chemical facility. All other chemical reagents used were obtained from Sigma-Aldrich unless noted.

### Cell culture, transfection, and viability assay

Human HEK 293T, HeLa, U937, and Jurkat cells were obtained from the American Type Culture Collection (ATCC). Mouse cell lines B16F10, Hepa1-6, LLC1, MB49, KPC, CT26, EMT6, MC38, EL4, and 4T1 were also from the ATCC and kindly provided by J. Sui (National Institute of Biological Sciences, Beijing). The NCI-60 panel of 60 cancer cells was obtained from the Development Therapeutic Program at the National Cancer Institute (Bethesda, MD) and cultured following the instructions provided. HEK 293T, HeLa, EL4, MC38, LLC1, Hepa1-6, MB49, and KPC were grown in Dulbecco’s modified Eagle’s medium (DMEM) supplemented with 10% (*v*/*v*) fetal bovine serum (FBS) and 2 mmol/L l-glutamine. U937, HL-60, Jurkat, B16F10, CT26, EMT6, 4T1, and the NCI-60 panel of 60 cancer cells were grown in RPMI 1640 medium supplemented with 10% FBS and 2 mmol/L l-glutamine. All cells were grown at 37°C in a 5% CO_2_ incubator.

Transient transfection was performed using jet PRIME (polyplus transfection) following the manufacturer’s instructions. For stable expression, lentiviral plasmids containing the desired gene were transfected into 293T cells together with the packing plasmids pSPAX2 and pMD2G with a ratio of 5:3:2. The supernatants were collected 48 h after transfection and used to infect indicated cells for another 48 h in the presence of polybrene. Stable expression cells were sorted by flow cytometry (BD Biosciences FACS Aria II) or selected by puromycin or blasticidin.

Cell viability was measured by the ATP assay using the CellTiter-Glo^®^ Luminescent Cell Viability Assay kit (Promega).

### Immunoblotting

Cells were collected, lysed directly in SDS sample buffer (100 mmol/L Tris-HCl, pH 6.8, 2% SDS, 10% glycerol, 100 mmol/L DTT, 0.1% BPB), and boiled at 95°C for 10 min. Samples with equal amounts of protein were then separated by SDS-PAGE and Semi-Dry transferred to Western blot PVDF membranes. After blocking, the membranes were subjected to standard immunoblotting, and the blotting signals were visualized by enhanced chemiluminescence. Antibodies used were anti-caspase-2 (ab179520; 1:1000), anti-RAIDD (ab76465; 1:1000), anti-caspase-7 (#9492; 1:1000), anti-cleaved caspase-8 (#9496; 1:1000), anti-caspase-9 (#9508; 1:1000), anti-Bid (#2002; 1:1000), anti-caspase-3 (#9662; 1:1000), anti-Flag (F3165/M2; 1:2,500), and anti-tubulin (T5168; 1:1000). Horseradish peroxidase (HRP)-conjugated anti-mouse IgG (NA931) and HRP-conjugated anti-rabbit IgG (NA934) were used at 1:5,000.

### FACS-based genome-wide CRISPR-Cas9 screen

Human CRISPR knockout gRNA plasmid library (GeCKO v2) encompassing 123,411 different gRNAs was generated by the Zhang laboratory (Sanjana et al., 2014) and obtained from Addgene. Amplification of the library and preparation of the lentivirus were performed as previously described (Shi et al., 2015). To perform the screen, U937 cells stably expressing Cas9 and inducible PIDD1-CC-2A-mRuby3 were seeded in the 15-cm dish and a total of 5 × 10^7^ cells were infected with the gRNA lentivirus library at the MOI of 0.3. Twenty-four hours after infection, cells were re-seeded and selected with 1 µg/mL puromycin after another 24 h. Six days later, 3 × 10^7^ puromycin-resistant cells were left untreated as the control sample. About 2 × 10^8^ puromycin-resistant cells were treated overnight with 1 μg/mL doxycycline and sorted for ZsGreen/mRuby3-double positive cells (~1.4%) on a BD Biosciences FACSAria II Flow Cytometer. The sorted cells were cultured for about 1 week, followed by other rounds of doxycycline treatment until the percentage of ZsGreen/mRuby3-double positive cells reached more than 95%. The cells that survived multiple rounds of doxycycline treatment were sorted and recovered for expansion. The recovered cells, together with the control cells with no treatment, were subjected to DNA extraction (Shi et al., 2015). Amplification of the gRNAs sequences was performed by using a two-step PCR method as described in our recent publication (Shi et al., 2015). The fold change was calculated by comparing the frequency of each gRNA in the screen sample with that in the control sample.

Another genome-wide CRISPR-Cas9 screen with compound HUHS015-36 as the stimulus of cell death followed the similar procedure as described above for the PIDDosome activation-based genetic screen, except that another human Genome-Wide Reduced Double-gRNA library (#137999) was used for screen in the *CASP2*^−/−^*CASP3*^−/−^*GSDMD*^−/−^ (TKO) HeLa cells rescued with exogenous caspase-2 expression.

### Chemical screen

Drug-related Screening Library (25 × 384-well plate, more than 7,000 compounds) was used for the chemical screen in the TKO HeLa cells rescued with exogenous caspase-2 expression. Briefly, the TKO HeLa cells with exogenous caspase-2 (HeLa-caspase-2^+^) were seeded into 384-well plates and cultured overnight to reach nearly 90% confluency. The compounds were added onto the cells (0.5 μL per well) by using Tecan freedom EVO150. After treatment for 12 h, the cell viability was measured by the ATP assay. The TKO HeLa cells with no caspase-2 rescuing (HeLa-caspase-2^−^) were operated similarly and served as negative controls. Compound candidates were selected by comparing the cell viability in the screen group with that in the control group for each compound (the cutoff ratio between HeLa-caspase-2^−^ and HeLa-caspase-2^+^ was set to above 3 for the primary hits).

### Generation of CRISPR/Cas9 knockout cell lines

Generation of knockout cells by the CRISPR-Cas9 method was performed as previously described (Shi et al., 2015; Wang et al., 2020). In brief, PX458 or lentiGuide-Puro plasmids containing the gRNAs targeting indicated genes were transfected or infected into the cells. Three days later, GFP-positive cells were sorted into single clones on 96-well plates by flow cytometry. Single clones were screened and verified by sequencing and immunoblotting. Sequences of the gRNAs used are AGTACTCCGCTCACTTCGCC & GACCCAGGGAAACTCCTGTA for *RAIDD*, TCGGCCTCGTGGCCTAGCAC & AAAGAACTGGAATTTCGCTC for *CASP2*, TGAGTGCATCACAAACCTAC & CTCCCGCTTGGGAAGAATAG for *BID*, CTTGCTTTAGACGTGCAGCG & GCTACACACCTGCAAGCACG for *GSDMD*, ACTAATATAAACAGAAGGCG & AATGGCACAAACATTTGAAA for *CASP3*, CAGGTATGGGCGTTCGAAA & AAGAGGGACGGTACAAACG for *CASP7*, AATCTTCTCGACCGACACA & TCTGGTCTGAGCACCACTG for *CASP9*, ATGATCAGACAGTATCCCCG & GGAAACACAGTTATTCACAG for *CASP8*, GTTTCATCCAGGATCGAGCA for *BAX* & GCAGGTAGCCCAGGACACAG for *BAK*, CCGGCAGCAGCAGCCGATAG & CCACCACTTCACGGCAGCGC for *PIDD1*.

### Caspase-2 activation assays

To stimulate caspase-2 activation, PIDD1-CC controlled by a Tet-On inducible system was introduced into indicated cells. Briefly, PIDD1-CC-containing lentivirus was added into the host cells, and infection was performed by centrifugation at 800 ×*g* for 99 min at room temperature followed by incubation at 37°C in a 5% CO_2_ incubator. To stimulate caspase-2 activation by PIDDosome, Jurkat, HL-60, and U937 cells were treated with 1 µg/mL doxycycline, followed by incubation for the indicated time to activate the PIDDosome. To stimulate caspase-2 activation by HUHS015 and its derivatives, HeLa cells were treated with 20 µmol/L HUHS015 for 9 h or 5 h for HUHS015-36, while Jurkat cells were treated with 20 µmol/L HUHS015-36 for 8 h. Cell lysates were analyzed by standard immunoblotting.

### Chemical synthesis

All reactions were carried out under an atmosphere of nitrogen in flame-dried glassware with magnetic stirring unless otherwise indicated. Reagents and solvents were obtained from commercial suppliers and used without further purification. Solvents were dried by passage through an activated alumina column under argon. Liquids and solutions were transferred via syringe. All reactions were monitored by thin-layer chromatography with E. Merck silica gel 60 F254 pre-coated plates (0.25 mm). ^1^H and ^13^C NMR spectra were recorded on Varian Inova-400 or 500 spectrometers. Data for ^1^H NMR spectra are reported as follows with CDCl_3_ (7.26 ppm), CD_3_OD (3.31 ppm), or DMSO-d_6_ (2.50 ppm) as an internal standard: chemical shift (*δ* ppm), multiplicity (*s* = singlet, *d* = doublet, *t* = triplet, *q* = quartet, sept = septet, m = multiplet, br = broad), coupling constant J (Hz), and integration. Data for ^13^C NMR spectra are reported in terms of chemical shift (*δ* ppm) with CDCl_3_ (77.23 ppm), CD_3_OD (49.00 ppm), or DMSO-d_6_ (39.52 ppm) as an internal standard. Samples preparation and purity analysis were conducted on Waters HPLC (Column: XBridge C18, 5 μm, 19 × 150 mm) with 2998PDA and 3100MS detectors, and Waters UPLC (Column: BEH C18, 1.7 μm, 2.1 × 50 mm) with PDA and SQD MS detectors, using ESI as ionization. HRMS data were obtained on a Thermo Q Exactive mass spectrometer.

All new compounds were synthesized via condensation of mono- or di-substituted 2-hydeaziney-1H-benzimidazoles and β-keto-ester. The synthesis routes of compounds 36 and 53 are shown in detail. For compounds with phenyl substituents, synthesis is according to the same route as that for compound 36 using mono- or di-substituted 1,2-diaminobenzene as starting materials. For compounds with pyridine substituents, synthesis is according to the same route as that for compound 53 using substituted pyridine-diamine as starting materials.

### Microscopy imaging, flow cytometry, and qRT-PCR

To examine the apoptotic morphology, Jurkat cells were seeded into a 12-well plate (Nunc Products, Thermo Fisher Scientific Inc.) at about 60% confluency and subjected to indicated treatments. Static bright-field cell images were captured by using ZOE Fluorescent cell imager (BioRad). For flow cytometry analyses, Jurkat cells and HL-60 cells were treated as indicated. Cells were collected, washed twice with PBS, and stained using the annexin V-FITC/PI or annexin V-Alexa 647/PI Apoptosis Assay Kit (Abmaking) according to the manufacturer’s instructions. The stained cells were further analyzed on a BD FACS Aria III flow cytometer and data were processed using FlowJo software. qRT-PCR was performed as previously described (ref). The mRNA level of target genes was normalized to that of ACTB. The primers used for human *PIDD1* and *ACTB* are Primer 1 (forward: TGTTCGAGGGCGAAGAGTTC; reverse: TCCAGAGTGGTGGTCACGTA) and Primer 2 (forward: GGACCTGACTGACTACCTCAT; reverse: CGTAGCACAGCTTCTCCTTAAT), respectively.

### Recombinant protein expression and purification

For recombinant expression of PIDDosome complex, the plasmids for PIDD1-DD, RAIDD, and caspase-2-CARD were co-transformed into *E*. *coli* BL21 (DE3), and the bacteria were cultured in LB medium with appropriate antibiotics. Target protein expression was induced with 0.4 mmol/L isopropyl β-l-1-thiogalactopyranoside (IPTG) at 20°C for 20 h after OD_600_ reached 0.8. Cells were lysed in buffer A containing 20 mmol/L Tris-HCl (pH 8.0), 5% glycerol, and 20 mmol/L imidazole. 6× His-tagged PIDD1-DD forms a non-covalent ternary complex (PIDDosome) with RAIDD and caspase-2-CARD when co-expressed in bacteria, which was then purified by Ni^2+^ affinity chromatography in buffer A. The 6× His tag of PIDD1-DD was removed by overnight digestion with homemade HRV3C protease at 4°C. The tag-removed PIDDosome was further purified by HiTrap Q anion exchange and Superdex G200 gel-filtration chromatography. Recombinant caspase-3 C163A and caspase-7 C186A proteins as well as MBP-fused caspase-2 pro-domain were expressed and purified similarly as described above for the PIDDosome, except that the 6× His-tagged proteins were directly eluted from Ni^2+^ affinity chromatography and subjected to anion exchange and gel-filtration chromatography without removal of the 6× His tag. Recombinant BID fused with a N-terminal 6× His-SUMO tag were purified by Ni^2+^ affinity chromatography in buffer B containing 20 mmol/L Tris-HCl (pH 8.0), 200 mmol/L NaCl, 5% glycerol, 20 mmol/L imidazole, and 1 mmol/L Tris(2-carboxyethyl)phosphine (TCEP). The 6× His-SUMO tag was removed by overnight digestion with homemade ULP1 protease at 4°C. The tag-removed proteins were further purified by HiTrap Q anion exchange chromatography and Superdex G75 gel-filtration chromatography. All purified target proteins were concentrated and stored in buffer C containing 20 mmol/L Tris-HCl (pH 8.0), 150 mmol/L NaCl, and 1 mmol/L TCEP.

Purification of active caspase-2 (p19/p12 form) and caspase-8 (p18/p10 form) followed the same procedures as described above for caspase-3/7 mutant proteins. Purified active caspases were concentrated and stored in buffer D containing 20 mmol/L Tris-HCl (pH 8.0), 150 mmol/L NaCl, 5% glycerol, and 1 mmol/L TCEP.

For gel-filtration chromatography analyses of PIDDosome, the purified complex was loaded into a Superdex 200 10/300 GL column and eluted with buffer C. The eluted fractions were subjected to SDS-PAGE analyses.

## *In vitro* caspase cleavage assay

Cleavage of BID, caspase-3 C163A, and caspase-7 C186A by active caspases was performed in the buffer containing 50 mmol/L HEPES (pH 7.5), 150 mmol/L NaCl, 3 mmol/L EDTA, 0.005% (*v*/*v*) Tween-20, and 10 mmol/L DTT. 20 µmol/L substrate proteins were reacted with 0.5 µmol/L caspases at 25°C for 30 min except for the time-course cleavage assays. Caspase-2 and -8 cleavage of BID was carried out at 37°C to achieve a more efficient cleavage. The reaction was terminated by adding the SDS-loading buffer. The samples were subjected to SDS-PAGE analyses.

### MicroScale thermophoresis

MicroScale thermophoresis (MST) experiments were carried out at 25°C in the PBST Buffer (PBS with 0.05%, *v*/*v* Tween 20) by using a Monolith 2020 (TNG) (MM-208). Specifically, purified MBP-fused caspase-2 pro-domain WT or L150P/Y151H double mutant proteins were labeled by using the Monolith His-Tag Labeling Kit and RED-tris-NTA 2nd Generation kit. Each group of experiments contains 16 concentrations of the compound mixed with 50 nmol/L labeled proteins in the capillary thermophoresis tubes. The highest concentration of the compound was 0.5 mmol/L, and the concentration decreased by half sequentially. The temperature-induced changes in fluorescence intensity of each sample in the capillary thermophoresis tubes were recorded by the Monolith instrument. The data were analyzed using the program provided by the manufacturer. The binding constant (*K*_D_) was calculated directly from the fitting.

## Supplementary Material

pwae020_suppl_Supplementary_Figures_S1-S5

## Data Availability

All data associated with this study are presented in the paper or the Supplementary Materials. The materials generated in this study are available from the corresponding author under an MTA.

## References

[CIT0001] Ahmad M , Srinivasula SM, Wang L et al CRADD, a novel human apoptotic adaptor molecule for caspase-2, and FasL/tumor necrosis factor receptor-interacting protein RIP. Cancer Res 1997;57:615–619. 9044836

[CIT0002] Aral K , Aral CA, Kapila Y. The role of caspase-8, caspase-9, and apoptosis inducing factor in periodontal disease. J Periodontol 2019;90:288–294. 30311940 10.1002/JPER.17-0716

[CIT0003] Beck H , Harter M, Hass B et al Small molecules and their impact in drug discovery: a perspective on the occasion of the 125th anniversary of the Bayer Chemical Research Laboratory. Drug Discov Today 2022;27:1560–1574. 35202802 10.1016/j.drudis.2022.02.015

[CIT0004] Bergeron L , Perez GI, Macdonald G et al Defects in regulation of apoptosis in caspase-2-deficient mice. Genes Dev 1998;12:1304–1314. 9573047 10.1101/gad.12.9.1304 PMC316779

[CIT0005] Bonzon C , Bouchier-Hayes L, Pagliari LJ et al Caspase-2-induced apoptosis requires bid cleavage: a physiological role for bid in heat shock-induced death. Mol Biol Cell 2006;17:2150–2157. 16495337 10.1091/mbc.E05-12-1107 PMC1446087

[CIT0006] Boucher D , Blais V, Drag M et al Molecular determinants involved in activation of caspase 7. Biosci Rep 2011;31:283–294. 20942802 10.1042/BSR20100111 PMC4485920

[CIT0007] Bouchier-Hayes L , Green DR. Caspase-2: the orphan caspase. Cell Death Differ 2012;19:51–57. 22075987 10.1038/cdd.2011.157 PMC3252831

[CIT0008] Bratton SB , Salvesen GS. Regulation of the Apaf-1-caspase-9 apoptosome. J Cell Sci 2010;123:3209–3214. 20844150 10.1242/jcs.073643 PMC2939798

[CIT0009] Brown-Suedel AN , Bouchier-Hayes L. Caspase-2 substrates: to apoptosis, cell cycle control, and beyond. Front Cell Dev Biol 2020;8:610022. 33425918 10.3389/fcell.2020.610022 PMC7785872

[CIT0010] Burigotto M , Mattivi A, Migliorati D et al Centriolar distal appendages activate the centrosome-PIDDosome-p53 signalling axis via ANKRD26. EMBO J 2021;40:e104844. 33350486 10.15252/embj.2020104844 PMC7883297

[CIT0012] Dickens LS , Boyd RS, Jukes-Jones R et al A death effector domain chain DISC model reveals a crucial role for caspase-8 chain assembly in mediating apoptotic cell death. Mol Cell 2012;47:291–305. 22683266 10.1016/j.molcel.2012.05.004 PMC3477315

[CIT0011] Di Donato N , Jean YY, Maga AM et al Mutations in CRADD result in reduced Caspase-2-mediated neuronal apoptosis and cause megalencephaly with a rare lissencephaly variant. Am J Hum Genet 2016;99:1117–1129. 27773430 10.1016/j.ajhg.2016.09.010 PMC5097945

[CIT0013] Enoksson M , Robertson JD, Gogvadze V et al Caspase-2 permeabilizes the outer mitochondrial membrane and disrupts the binding of cytochrome c to anionic phospholipids. J Biol Chem 2004;279:49575–49578. 15475367 10.1074/jbc.C400374200

[CIT0014] Fava LL , Bock FJ, Geley S et al Caspase-2 at a glance. J Cell Sci 2012;125:5911–5915. 23447670 10.1242/jcs.115105

[CIT0015] Fava LL , Schuler F, Sladky V et al The PIDDosome activates p53 in response to supernumerary centrosomes. Genes Dev 2017;31:34–45. 28130345 10.1101/gad.289728.116 PMC5287111

[CIT0016] Guo Y , Srinivasula SM, Druilhe A et al Caspase-2 induces apoptosis by releasing proapoptotic proteins from mitochondria. J Biol Chem 2002;277:13430–13437. 11832478 10.1074/jbc.M108029200

[CIT0017] Ho LH , Taylor R, Dorstyn L et al A tumor suppressor function for caspase-2. Proc Natl Acad Sci U S A 2009;106:5336–5341. 19279217 10.1073/pnas.0811928106 PMC2664004

[CIT0018] Janssens S , Tinel A. The PIDDosome, DNA-damage-induced apoptosis and beyond. Cell Death Differ 2012;19:13–20. 22095286 10.1038/cdd.2011.162 PMC3252840

[CIT0019] Kuida K. Caspase-9. Int J Biochem Cell Biol 2000;32:121–124. 10687948 10.1016/s1357-2725(99)00024-2

[CIT0020] Kuida K , Haydar TF, Kuan CY et al Reduced apoptosis and cytochrome c-mediated caspase activation in mice lacking caspase 9. Cell 1998;94:325–337. 9708735 10.1016/s0092-8674(00)81476-2

[CIT0021] Li H , Zhu H, Xu CJ et al Cleavage of BID by caspase 8 mediates the mitochondrial damage in the Fas pathway of apoptosis. Cell 1998;94:491–501. 9727492 10.1016/s0092-8674(00)81590-1

[CIT0022] Lin Y , Ma W, Benchimol S. Pidd, a new death-domain-containing protein, is induced by p53 and promotes apoptosis. Nat Genet 2000;26:122–127. 10973264 10.1038/79102

[CIT0023] Manzl C , Peintner L, Krumschnabel G et al PIDDosome-independent tumor suppression by Caspase-2. Cell Death Differ 2012;19:1722–1732. 22595758 10.1038/cdd.2012.54 PMC3438502

[CIT0024] McIlwain DR , Berger T, Mak TW. Caspase functions in cell death and disease. Cold Spring Harb Perspect Biol 2013;5:a008656. 23545416 10.1101/cshperspect.a008656 PMC3683896

[CIT0025] O’Reilly LA , Ekert P, Harvey N et al Caspase-2 is not required for thymocyte or neuronal apoptosis even though cleavage of caspase-2 is dependent on both Apaf-1 and caspase-9. Cell Death Differ 2002;9:832–841. 12107826 10.1038/sj.cdd.4401033

[CIT0026] Park HH , Logette E, Raunser S et al Death domain assembly mechanism revealed by crystal structure of the oligomeric PIDDosome core complex. Cell 2007;128:533–546. 17289572 10.1016/j.cell.2007.01.019 PMC2908332

[CIT0027] Paroni G , Henderson C, Schneider C et al Caspase-2 can trigger cytochrome C release and apoptosis from the nucleus. J Biol Chem 2002;277:15147–15161. 11823470 10.1074/jbc.M112338200

[CIT0028] Parsons MJ , McCormick L, Janke L et al Genetic deletion of caspase-2 accelerates MMTV/c-neu-driven mammary carcinogenesis in mice. Cell Death Differ 2013;20:1174–1182. 23645210 10.1038/cdd.2013.38 PMC3741497

[CIT0029] Poreba M , Rut W, Groborz K et al Potent and selective caspase-2 inhibitor prevents MDM-2 cleavage in reversine-treated colon cancer cells. Cell Death Differ 2019;26:2695–2709. 30976094 10.1038/s41418-019-0329-2 PMC7224280

[CIT0030] Puccini J , Shalini S, Voss AK et al Loss of caspase-2 augments lymphomagenesis and enhances genomic instability in Atm-deficient mice. Proc Natl Acad Sci U S A 2013;110:19920–19925. 24248351 10.1073/pnas.1311947110 PMC3856814

[CIT0031] Rodriguez J , Lazebnik Y. Caspase-9 and APAF-1 form an active holoenzyme. Genes Dev 1999;13:3179–3184. 10617566 10.1101/gad.13.24.3179 PMC317200

[CIT0032] Sanjana NE , Shalem O, Zhang F. Improved vectors and genome-wide libraries for CRISPR screening. Nat Methods 2014;11:783–784. 25075903 10.1038/nmeth.3047 PMC4486245

[CIT0033] Schleich K , Krammer PH, Lavrik IN. The chains of death: a new view on caspase-8 activation at the DISC. Cell Cycle 2013;12:193–194. 23287476 10.4161/cc.23464 PMC3575441

[CIT0034] Shalini S , Dorstyn L, Wilson C et al Impaired antioxidant defence and accumulation of oxidative stress in caspase-2-deficient mice. Cell Death Differ 2012;19:1370–1380. 22343713 10.1038/cdd.2012.13 PMC3392626

[CIT0035] Sheikh TI , Vasli N, Pastore S et al Biallelic mutations in the death domain of PIDD1 impair caspase-2 activation and are associated with intellectual disability. Transl Psychiatry 2021;11:1. 33414379 10.1038/s41398-020-01158-w PMC7791037

[CIT0036] Shi J , Zhao Y, Wang K et al Cleavage of GSDMD by inflammatory caspases determines pyroptotic cell death. Nature 2015;526:660–665. 26375003 10.1038/nature15514

[CIT0037] Stennicke HR , Jurgensmeier JM, Shin H et al Pro-caspase-3 is a major physiologic target of caspase-8. J Biol Chem 1998;273:27084–27090. 9765224 10.1074/jbc.273.42.27084

[CIT0039] Tinel A , Tschopp J. The PIDDosome, a protein complex implicated in activation of caspase-2 in response to genotoxic stress. Science 2004;304:843–846. 15073321 10.1126/science.1095432

[CIT0038] Tinel A , Janssens S, Lippens S et al Autoproteolysis of PIDD marks the bifurcation between pro-death caspase-2 and pro-survival NF-kappaB pathway. EMBO J 2007;26:197–208. 17159900 10.1038/sj.emboj.7601473 PMC1782377

[CIT0040] Tu S , McStay GP, Boucher LM et al *In situ* trapping of activated initiator caspases reveals a role for caspase-2 in heat shock-induced apoptosis. Nat Cell Biol 2006;8:72–77. 16362053 10.1038/ncb1340

[CIT0041] Twiddy D , Cain K. Caspase-9 cleavage, do you need it? Biochem J 2007;405:e1–e2. 17555401 10.1042/BJ20070617 PMC1925255

[CIT0042] Uctepe E , Vona B, Esen FN et al Bi-allelic truncating variants in CASP2 underlie a neurodevelopmental disorder with lissencephaly. Eur J Hum Genet 2024;32:52–60. 37880421 10.1038/s41431-023-01461-2 PMC10772072

[CIT0043] Upton JP , Austgen K, Nishino M et al Caspase-2 cleavage of BID is a critical apoptotic signal downstream of endoplasmic reticulum stress. Mol Cell Biol 2008;28:3943–3951. 18426910 10.1128/MCB.00013-08 PMC2423129

[CIT0044] Van Opdenbosch N , Lamkanfi M. Caspases in cell death, inflammation, and disease. Immunity 2019;50:1352–1364. 31216460 10.1016/j.immuni.2019.05.020 PMC6611727

[CIT0045] Varfolomeev EE , Schuchmann M, Luria V et al Targeted disruption of the mouse Caspase 8 gene ablates cell death induction by the TNF receptors, Fas/Apo1, and DR3 and is lethal prenatally. Immunity 1998;9:267–276. 9729047 10.1016/s1074-7613(00)80609-3

[CIT0046] Wagner KW , Engels IH, Deveraux QL. Caspase-2 can function upstream of bid cleavage in the TRAIL apoptosis pathway. J Biol Chem 2004;279:35047–35052. 15173176 10.1074/jbc.M400708200

[CIT0047] Wang K , Sun Q, Zhong X et al Structural mechanism for GSDMD targeting by autoprocessed caspases in pyroptosis. Cell 2020;180:941–955.e20. 32109412 10.1016/j.cell.2020.02.002

[CIT0048] Zaki MS , Accogli A, Mirzaa G et al Pathogenic variants in PIDD1 lead to an autosomal recessive neurodevelopmental disorder with pachygyria and psychiatric features. Eur J Hum Genet 2021;29:1226–1234. 34163010 10.1038/s41431-021-00910-0 PMC8385073

[CIT0049] Zhang Y , Li W, Laurent T et al Small molecules, big roles—the chemical manipulation of stem cell fate and somatic cell reprogramming. J Cell Sci 2012;125:5609–5620. 23420199 10.1242/jcs.096032 PMC4067267

[CIT0050] Zheng TS , Hunot S, Kuida K et al Caspase knockouts: matters of life and death. Cell Death Differ 1999;6:1043–1053. 10578172 10.1038/sj.cdd.4400593

