# Supplementary material for: Dissecting caspase-2-mediated cell death: from intrinsic PIDDosome activation to chemical modulation
Source: Protein Cell. 2024 Apr 27;15(12):889–905. doi: 10.1093/procel/pwae020 (PMC11637483; doi:10.1093/procel/pwae020)
Supplement: pwae020_suppl_Supplementary_Figures_S1-S5 [file pwae020_suppl_supplementary_figures_s1-s5.pdf]

Supplementary Figures for

Dissecting caspase-2-mediated cell death: from intrinsic PIDDosome activation to chemical modulation

Mengxue Zeng, Kun Wang, Qingcui Wu, Jingjin Ding, Dan Xie, Xiangbing Qi, and Feng Shao

This file contains: Figure S1 to S5

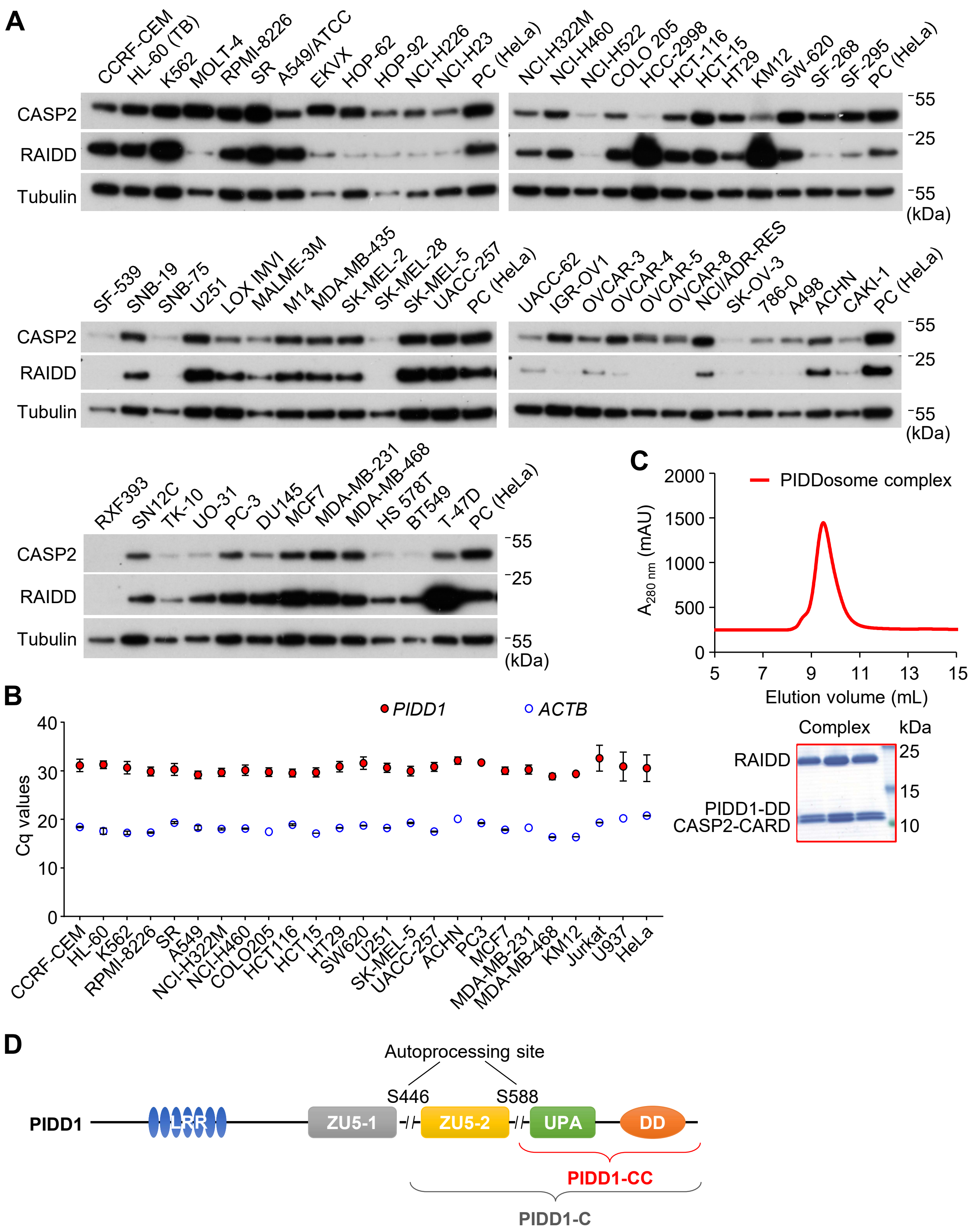

Figure S1. Profiling endogenous caspase-2 and RAIDD expression in the NCI-60 and biochemical characterization of the PIDDosome complex.

(A) Lysates of 60 cancer cell lines from the NCI-60 (a panel of 60 diverse human cancer cell lines used by the US National Cancer Institute to screen and evaluate anti-cancer drugs) were analyzed by immunoblotting using indicated antibodies. Wild-type HeLa cell lysate was included as a positive (PC). (B) *PIDD1* expression was profiled by quantitative real time reverse transcription polymerase chain reaction (qRT-PCR) in indicated cell lines. *ACTB* expression was measured in parallel as a constitutive reference with high expression. (C) Gel-filtration chromatography analysis of the PIDDosome complex formed by PIDD1 death domain (PIDD1-DD), full-length RAIDD and caspase-2 CARD domain (CASP2-CARD), along with SDS-PAGE analysis of the complex in the elution peak fractions. (D) Schematic diagram of PIDD1 domain organization. Data in (A−C) are representative of three independent experiments.

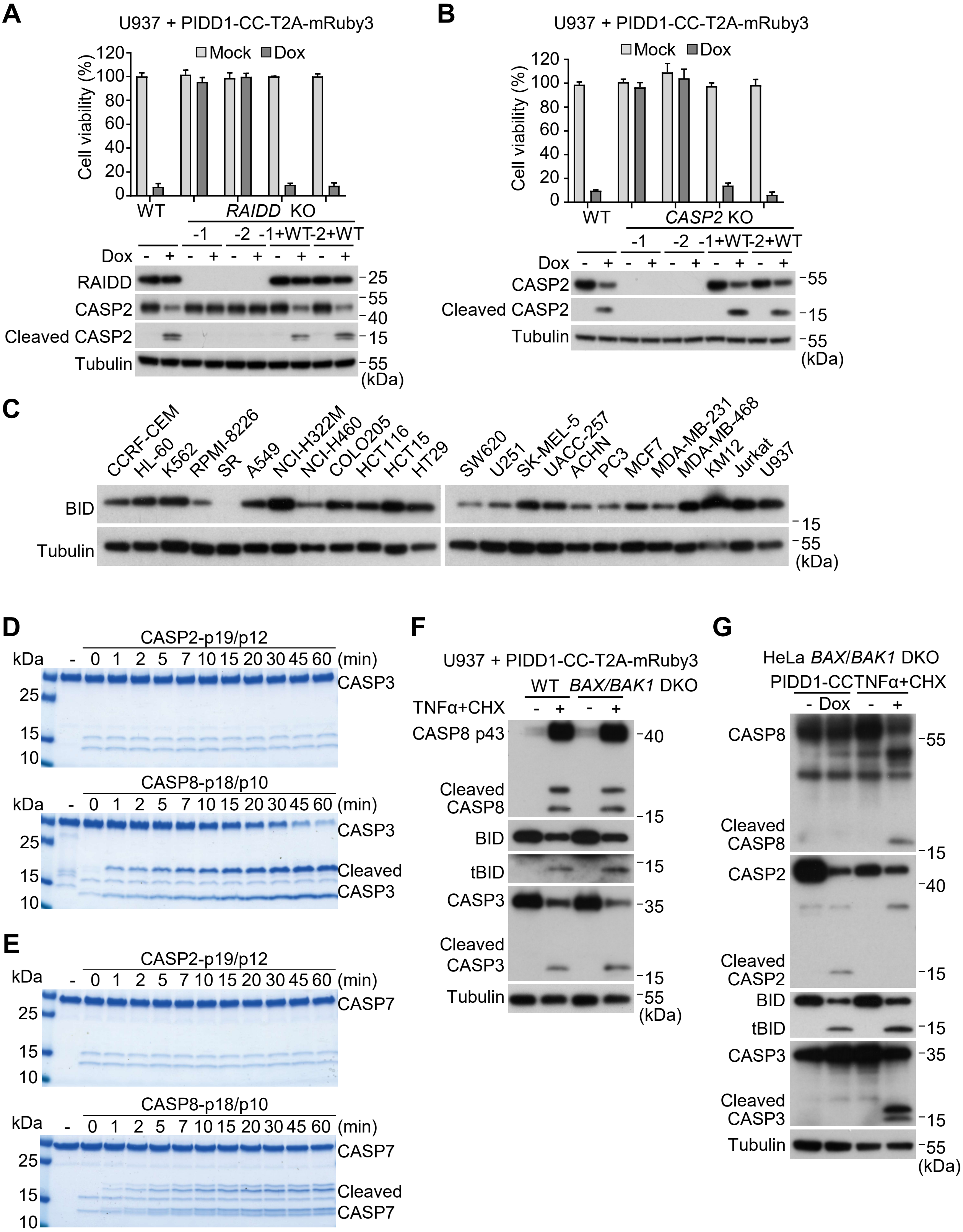

A
U937 + PIDD1-CC-T2A-mRuby3
Mock
Dox
Cell viability (%)
WT
RAIDD KO
-1
-2
-1+WT
-2+WT
RAIDD
CASP2
Cleaved CASP2
Tubulin
(kDa)
B
U937 + PIDD1-CC-T2A-mRuby3
CASP2 KO
C
CCRF-CEM
HL-60
K562
RPMI-8226
SR
A549
NCI-H322M
NCI-H460
COLO205
HCT116
HCT15
HT29
SW620
U251
SK-MEL-5
UACC-257
ACHN
PC3
MCF7
MDA-MB-231
MDA-MB-468
KM12
Jurkat
U937
BID
Tubulin
D
CASP2-p19/p12
CASP8-p18/p10
CASP3
Cleaved CASP3
E
CASP7
Cleaved CASP7
F
U937 + PIDD1-CC-T2A-mRuby3
WT
BAX/BAK1 DKO
TNFα+CHX
CASP8 p43
Cleaved CASP8
tBID
G
HeLa BAX/BAK1 DKO
PIDD1-CC
CASP8

Figure S2

Figure S2. Profiling the differences between caspase-2 and caspase-8 in BID and caspase-3 processing. (A and B) RAIDD and CASP2 are both essential for PIDD1-CC-induced caspase-2 activation and cell death in U937 cells. Two *RAIDD*$^{-/-}$ (KO) clones (A) and two *CASP2*$^{-/-}$ (KO) clones (B) of U937 cells, as well as the knockout clones complemented with wild-type (WT) RAIDD and caspase-2, respectively, were stimulated by dox-induced expression of PIDD1-CC for 11 h. ATP-based cell viability is expressed as mean ± s.d. from three technical replicates. Immunoblotting detecting caspase-2 processing is shown. (C) Immunoblots of endogenous BID expression in the 22 RAIDD/CASP2 double-positive cancer cell lines assayed in Fig. 1A. (D and E) *In vitro* cleavage of caspase-3/7 by active form of caspase-8 but not caspase-2. Purified caspase-3-C163A (D) or caspase-7-C186A (E) proteins were incubated with active caspase-2 and caspase-8 proteins for indicated time, and the samples were subjected to SDS-PAGE analysis. (F) Validation of the roles of BAX and BAK in caspase-8-mediated BID and caspase-3 processing by immunoblotting. WT and *BAX*$^{-/-}$*BAK*$^{-/-}$ (DKO) U937 cells were treated with TNFα plus cycloheximide (CHX) for 2 h to activate caspase-8. (G) Differential roles of BAX and BAK in caspase-2 and caspase-8 activation-mediated processing of BID and caspase-3. WT and *BAX*$^{-/-}$*BAK*$^{-/-}$ (DKO) HeLa cells were stimulated by dox-induced expression of PIDD1-CC for caspase-2 activation or TNFα plus CHX treatment for caspase-8 activation. Processing of caspase-2, -3, and -8, and BID was analyzed by immunoblotting. All data are representative of three independent experiments.

Gene	gRNA	FC_recover	Ranking
BID	BID_4	771.5143	1
BID	BID_1	765.6589	2
BID	BID_6	748.5108	3
BID	BID_5	747.0161	4
BID	BID_3	507.1263	6
BID	BID_2	359.9454	11
CASP2	CASP2_2	523.174	5
CASP2	CASP2_3	499.6242	7
CASP2	CASP2_1	387.4627	10
CASP2	CASP2_6	239.9944	13
CASP2	CASP2_5	152.3754	15
CRADD	CRADD_6	423.511	8
CRADD	CRADD_2	404.5781	9
CRADD	CRADD_1	257.0858	12
CRADD	CRADD_3	235.3633	14
CRADD	CRADD_4	25.89795	19
DDIT4	DDIT4_6	25.65607	88
DDIT4	DDIT4_2	10.56757	430
DDIT4	DDIT4_4	8.342154	710
DDIT4	DDIT4_5	7.615992	845
DDIT4	DDIT4_1	5.279328	1615
DET1	DET1_6	9.074155	596
DET1	DET1_5	7.724657	817
DET1	DET1_4	7.398984	897
DET1	DET1_2	5.594437	1462
FLCN	FLCN_2	13.99241	247
FLCN	FLCN_3	10.46674	443
FLCN	FLCN_6	8.373865	701
FLCN	FLCN_5	7.964379	764
LAMTOR2	LAMTOR2_5	9.797291	504
LAMTOR2	LAMTOR2_6	7.710842	820
LAMTOR2	LAMTOR2_3	7.058851	986
LAMTOR2	LAMTOR2_2	6.09096	1273
PTBP1	PTBP1_3	106.188	24
PTBP1	PTBP1_2	25.52036	89
PTBP1	PTBP1_5	13.61567	264
PTBP1	PTBP1_4	8.184038	725
PTBP1	PTBP1_1	7.963278	765
RRAGA	RRAGA_3	10.39173	450
RRAGA	RRAGA_2	6.674335	1093
RRAGA	RRAGA_6	5.32618	1593
RRAGA	RRAGA_1	5.255568	1629
RRAGA	RRAGA_5	4.972998	1795

Figure S3. CRISPR-Cas9 screen of PIDD1-CC-induced cell death.

U937 cells harboring PIDD1-CC-T2A-mRuby3 and ZsGreen were treated with doxycycline to induce PIDDosome-stimulated apoptosis. The pool of RFP/GFP-double-positive cells were subjected to gRNA sequencing to identify enriched clones. Among the top 2,000 gRNA hits, 9 genes were hit by four or more gRNAs, but only RAIDD, CASP2, and BID passed subsequent validation using their knockout cell lines.

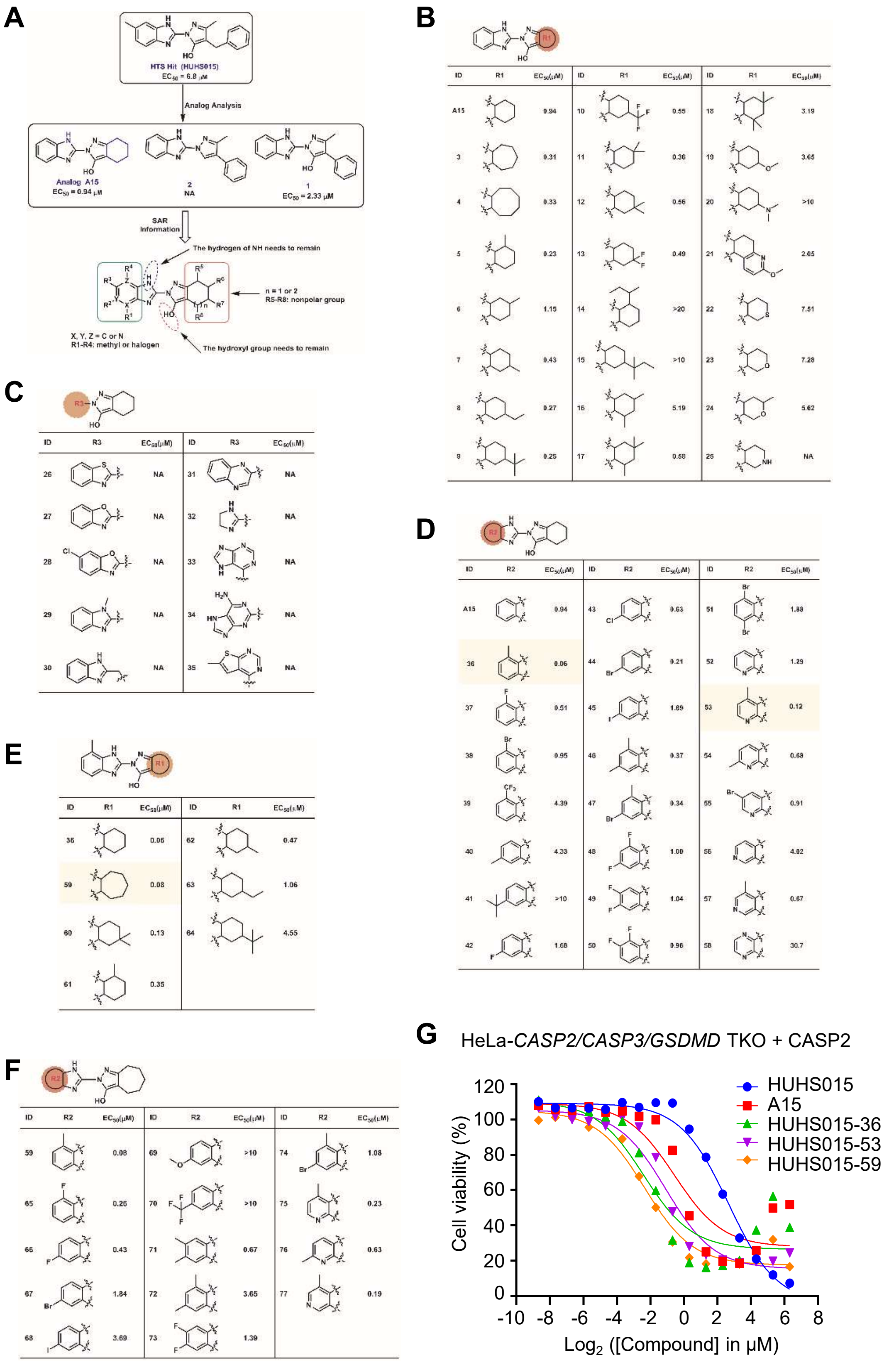
A
HTS Hit (HUHS015)
EC50 = 6.8 μM
Analog Analysis
Analog A15
EC50 = 0.94 μM
2
NA
1
EC50 = 2.33 μM
SAR Information
The hydrogen of NH needs to remain
n = 1 or 2
R5-R8: nonpolar group
X, Y, Z = C or N
R1-R4: methyl or halogen
The hydroxyl group needs to remain
B
C
D
E
F
G
HeLa-CASP2/CASP3/GSDMD TKO + CASP2
HUHS015
A15
HUHS015-36
HUHS015-53
HUHS015-59
Cell viability (%)
Log2 ([Compound] in μM)

Figure S4

Figure S4. Structure-Activity Relationship analyses of HUHS015 and its derivatives.

(A) Structure-Activity Relationship (SAR) information of HUHS015 from its analog A15 and designed derivatives by extensive chemical modifications. (B−F) The chemical structures and potencies of A15 and its derivatives designed by SAR-guided modifications. The modifications include alterations in the ring structure fused with the hydroxylpyrazole moiety (B), replacing the benzimidazole moiety with other types of heterocycles (C), substitutions and heteroatom incorporations in the phenyl group of the benzimidazole moiety (D), alterations in the ring structure fused with the hydroxylpyrazole moiety of compound 36 (E), substitutions and heteroatom incorporations in the phenyl group of the benzimidazole moiety of compound 59 (F). The EC_{50} of all designed compounds are listed. (G) EC_{50} calculation plots for HUHS015, A15 and representative derivatives are shown.

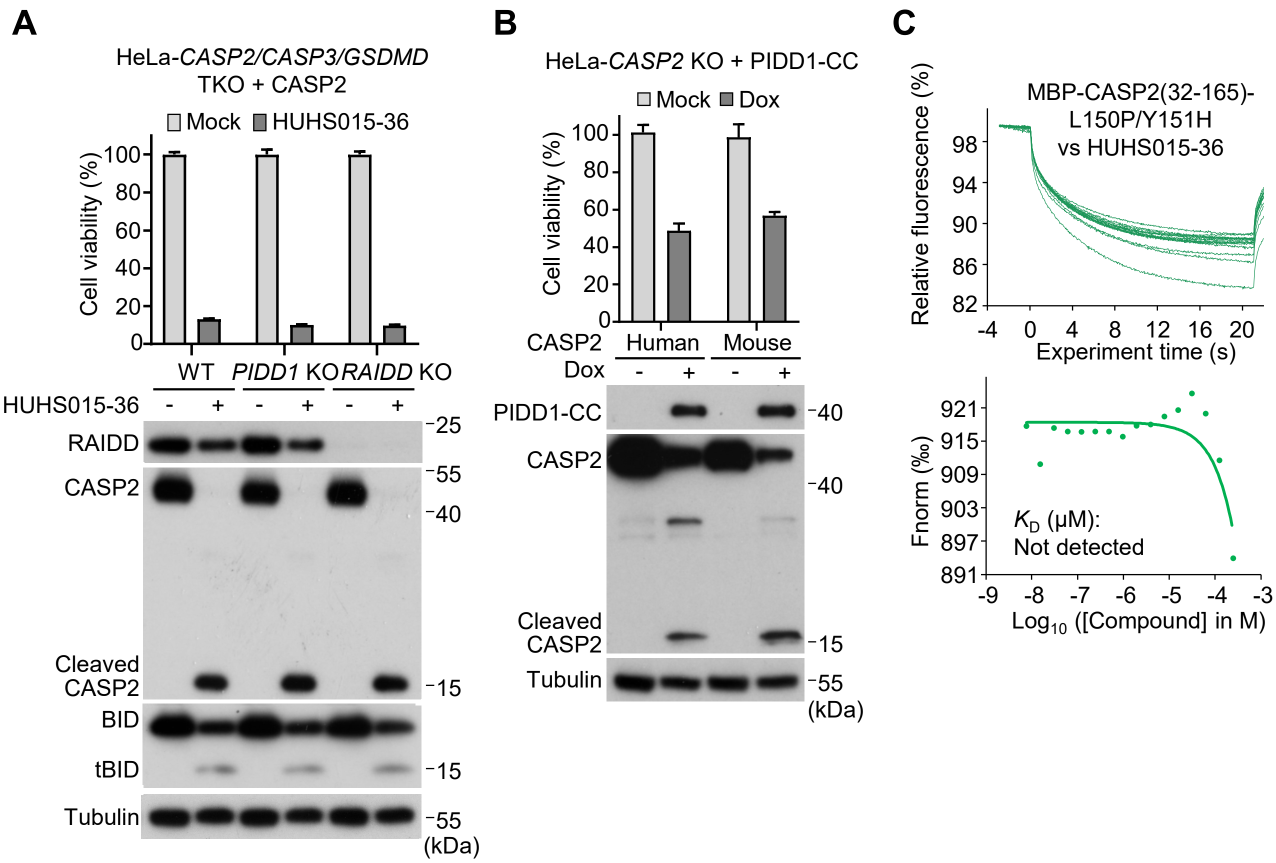

Figure S5. HUHS015-derived compound activates human caspase-2 in a different way from that for PIDDosome-mediated caspase-2 activation.

(A) HUHS015-36 activates caspase-2 independently of PIDDosome. WT, *PIDD1*$^{-/-}$ (KO) and *RAIDD*$^{-/-}$ (KO) HeLa cells with exogenous caspase-2 expression were treated with HUHS015-36 for 5 h. ATP-based cell viability is expressed as mean ± s.d. from three technical replicates. Immunoblotting detecting caspase-2 and BID processing is shown. (B) PIDDosome activates both human and mouse caspase-2 to induce cell death. *CASP2*$^{-/-}$ (KO) HeLa cells rescued with human or mouse caspase-2 expression were stimulated by dox-induced expression of PIDD1-CC. ATP-based cell viability is expressed as mean ± s.d. from three technical replicates. Immunoblotting detecting PIDD1-CC expression and caspase-2 processing is shown. (C) Microscale thermophoresis (MST) detects no binding between HUHS015-36 and MBP-fused human caspase-2 pro-domain containing L150P/Y151H double mutations. MST profiles for raw binding signals and affinity (K_D) measurement are shown. All data are representative of three independent experiments.
